# Molecular Design and Spodumene Flotation—A Review

**DOI:** 10.3390/ijms25063227

**Published:** 2024-03-12

**Authors:** Jose I. Retamal, Pedro A. Robles, Gonzalo R. Quezada, Ricardo I. Jeldres

**Affiliations:** 1 Escuela de Ingeniería Química, Pontificia Universidad Católica de Valparaíso, Valparaíso 2340000, Chile; jr.lecaros23@gmail.com; 2 Escuela de Ingeniería Química, Universidad del Bio-Bio, Concepción 4030000, Chile; grquezada@ubiobio.cl; 3 Departamento de Ingeniería Química y Procesos de Minerales, Facultad de Ingeniería, Universidad de Antofagasta, Antofagasta 1240000, Chile; ricardo.jeldres@uantof.cl

**Keywords:** spodumene, flotation, molecular dynamics, lithium extraction, collector, Gromacs, Materials Studio

## Abstract

Spodumene flotation stands as the most commonly used method to concentrate lithium minerals. However, it faces significant challenges related to low collector recoveries and similarity in the surface characteristics of the minerals, which make the effective separation of this valuable mineral difficult. For this reason, numerous researchers have conducted studies to address and confront this problem. In this work, an exhaustive bibliographic search was carried out using keywords and search queries, and the results were structured in three sections according to temporal, methodological, and thematic criteria. The first section covers the period from 1950 to 2004, focusing on experimental tests. The second section covers from 2004 to the present and focuses on flotation tests and measurement analysis. Simultaneously, the third section spans from 2011 to the present and is based on molecular dynamics simulations. Topics covered include spodumene surface properties, the influence of metal ions, pre-treatment techniques, and the use of collectors. Ultimately, molecular dynamics simulations are positioned as a tool that accurately represents experimental phenomena. In this context, specialized software such as Materials Studio or Gromacs prove to be reliable instruments that allow a detailed study of mineral surfaces and other elements to be carried out, which justifies their consideration for future research in this scientific field.

## 1. Introduction

Lithium has great economic interest due to its properties, such as high reactivity, flammability, and electrochemical potential [1,2]. These qualities led to its use as an energy element in manufacturing batteries for electrical appliances such as cars, portable devices, or energy storage systems. However, it has also been used for other industries, e.g., glass manufacturing, ceramics, lubricants, and metallurgical industries. Its main use is for the manufacture of lithium batteries, with 56% of lithium used for this purpose, followed by the ceramic and glass industry, with 23% [3,4,5].

There are 86 million metric tons of lithium resources worldwide, most of which correspond to brines and pegmatite rocks. Among the countries with the largest reserves are Argentina, Bolivia, Chile, Australia, and the United States [6]. Lithium extraction from brines is carried out by adsorption, precipitation, ion exchange, or solvent extraction. The recovery percentage depends mainly on the composition, volume, and accessibility of the resource [3,7]. Meanwhile, lithium pegmatites contain minerals such as spodumene, petalite, lepidolite, amblygonite, zinnwaldite, triphylite, and eucryptite. Of these, spodumene is the most important economically due to its composition having the highest theoretical percentage of lithium [5,8].

Lithium recovery from this type of mineral is usually achieved through gravity separation, magnetic separation, and foam flotation [5]. Gravity separation is used to separate the ore of interest from the gangue. This is accomplished by utilizing the difference in density between spodumene and other minerals, such as quartz, albite, or muscovite. Magnetic separation is used before flotation to remove many iron-containing minerals. This is achieved through industrial magnets installed on the belts used to transport the minerals. Finally, foam flotation is utilized to separate minerals by using the difference in the surface properties of the minerals to recover the valuable material from the gangue [1,5,9,10]. This is one of the most common methods of mineral separation currently in use [11,12].

Albeit the most common method, care must be taken in the different factors that impact and determine flotation performance. These factors include mineral surface chemistry, collector type, pre-treatment techniques, and reagents. In the case of lithium flotation, there is a great similarity in the mineralogical characteristics between the ores that contain lithium, e.g., the isoelectric point (IEP), which are similar values of less than three between lithium minerals and gangue minerals, resulting in similar properties that make selective flotation complex to perform [5,9]. In addition, a low selectivity by the fatty acid collectors has been detected, commonly used in salt as sodium oleate (NaOL), due to poor solubility and sensitivity to changes in pH and concentration [11]. To improve flotation performance, reagents are added that allow the addition of certain ions that activate the spodumene surface and increase its floatability. Although good results have been observed, it remains a debate which ions should be added, the dosage, the collector that benefits most from this activation, and the associated interaction mechanism between the ion and the surface of the mineral [13].

For this reason, research has mainly focused on factors that determine the chemical characteristics of the mineral surface, such as particle size, isomorphic substitution, grinding types, wetting, anisotropic properties, and surface crystal chemistry [14,15,16,17,18,19,20].

One of the most frequently used collectors is NaOL, and its research continues. Studies were carried out on mixtures of collectors that increase flotation performance and synthesis of new collectors. Different authors mixed anionic collectors with cationic collectors to evaluate their selective capacity. They also investigated the adsorption mechanisms of these collectors with zeta potential measurements, analysis of Fourier transform infrared spectroscopy (FTIR), and X-ray photoelectron spectroscopy (XPS), finding good separation results; however, these require further studies before being taken to an industrial level [21,22,23,24,25]. By contrast, other authors have synthesized new collectors, obtaining good flotation results, but it remains unclear what the adsorption mechanism is on the surface of the mineral [26,27]. 

Finally, the addition of activators that react with the mineral’s surface and increase the collector’s adsorption was also the subject of research. Metal ions, such as Fe^3+^, Ca^2+^, Mg^2+^, Cu^2+^, and Pb^2+^, and their adsorption mechanisms in the mineral surface were studied, presenting a significant effect on the adsorption of the collector at the surface of the mineral. Those that favor flotation the most include iron, calcium, and magnesium ions [28,29,30,31,32].

Without an exact understanding of how the interaction mechanisms work, some researchers have relied on computational software based on quantum chemistry, density functional theory, and molecular dynamics simulations to support the experimental testing and the measurements they perform. These computational tools help analyze the interaction between atoms of the different surfaces of the minerals with collectors or metal ions, delivering relevant flotation data from an atomic and electronic perspective [33,34,35].

In this review, after the bibliographic search, the research was organized into three sections according to the methodology used. First, older research mainly used flotation tests to study performance and different process parameters. Secondly, there is research that used experimental tests relying on other analysis techniques to study the surface characteristics of spodumene, the effect of metal ions, and treatment methods to study different collectors. Finally, a section is dedicated to research that mainly utilizes computational tools and simulations to analyze the interaction between the surface of the ore with collectors and other elements. All analysis was summarized, indicating the topic, methodology, results, and most relevant conclusions. This review aims to guide how research on spodumene flotation has been carried out to identify gaps or research opportunities that will improve the process. In addition, it provides a new perspective from the standpoint of molecular dynamics simulations to be used to analyze the interaction between the surface of spodumene and other elements at the molecular level.

## 2. Relevant Sections

To conduct the bibliographic search, the database “Web of Science” was utilized, using keywords such as spodumene flotation, lithium recovery, surface characteristics of spodumene, spodumene molecular design, effect of metal ions on spodumene, and molecular dynamics of spodumene. The search results are presented in Table 1 below.

From the first search, a total of 6337 scientific articles were found. The relevant keywords were used to build search queries to reduce this quantity and better focus the studied topics. It is important to mention that the keywords “Spodumene molecular design” was not utilized, as researchers use the term “molecular dynamics” to refer to molecular design simulations or molecular dynamics over “molecular design”. In this way, the search results using search queries are presented in Table 2.

By using search queries, the number of articles for the bibliographic review was greatly narrowed down, reducing the number of results to only 58.

Finally, the articles were sorted by year, theme, objective, methodology used, and results to establish their similarities and differences. For this reason, the publications were split into three sections, which are presented in Table 3.

In Table 3, it is possible to observe that the first section groups seven articles, ranging from the years 1953 to 2004. This section focuses on proposing process flow diagrams for lithium recovery from spodumene, presents relevant information on minerals and extraction methods, and uses primarily experimental flotation tests or batch flotation for this end. The second section groups 52 articles spanning from 2003 to the 2023. This section focuses on various factors influencing spodumene flotation. It analyzes the performance and behavior of mixed and new collectors, the effect of metal ions, the chemical characteristics of the mineral surface, pre-treatment techniques, etc. It primarily employs flotation tests and various measurement techniques to achieve its objective. Finally, the third section groups only three articles from 2011 to 2023. These articles focus on the interaction mechanism between the mineral and collectors or different elements in flotation. The uniqueness of this section is that they mostly or entirely use computational tools to study mechanisms at the molecular or atomic level.

### 2.1. First Years of Research (1953–2004)

The first scientific papers investigating spodumene flotation for the obtention of lithium were made in the United States more than 60 years ago. Without the technological development or measurement techniques that exist today, research was conducted with a purely experimental approach that sought to obtain the highest performance without going into greater detail about the mechanisms of this stage. 

In 1953, Mason K. Banks and his collaborators described the results of flotation tests performed in 1951, obtaining a concentration process that had not previously been recorded in literature. Using pilot-scale tests, different operational variables were studied, such as caustic soda dosage in the grinding stage before flotation, the pH of the Rougher and Cleaner cells, conditioning time, and the use of different reagents. It was concluded that achieving a 75% spodumene recovery is possible using caustic soda grinding, minerals containing at least 15% spodumene, maintaining a high pulp density, and avoiding the presence of hornblende [36]. 

Two years later, in South Dakota, research on this process continued. However, it was conducted theoretically, describing the deposits of that time, such as Edison, Mateen, Longview-Beecher, Etta, and Tinton. The research also mentions methods to achieve spodumene separation, such as manual sorting, separation by heavy means, or using different weights and flotation [37]. Despite not being an experimental investigation, the study provided relevant information on separating spodumene from gangue minerals, such as feldspar, quartz, and mica. Conversely, Colton compiled the main characteristics of lithium minerals, i.e., spodumene, zinnwaldite, and lepidolite, highlighting lithium as the most important ore. Similarly, compared to previous research, different separation methods are described. However, it also introduces unconventional flotation, where the mineral of interest sinks into the tailings. Consequently, the obtention of lithium carbonate through lithium chloride or hydroxides obtained from flotation was proposed [38]. 

Using the process above of spodumene separation, research focused on options to optimize the process using different reagents or procedures. In this line, McVay and his collaborators conducted experimental and batch flotation tests to determine optimal separation conditions. The use of other collectors, lignin dispersants, solids conditioning, and different pH of the pulp was studied. They concluded that a good spodumene grade can be obtained by using sodium fluoride and sodium lignin sulfonate in combination with oleic acid as a collector at pH 6.5–8 [39]. 

Research was carried out not only in the United States; in Brazil, a team of collaborators studied the cationic flotation of lithium minerals. Unlike previous authors, however, zeta potential measurements were included in addition to micro-flotation tests to study the interaction between the ore and the collectors. It was found that maximum floatability can be achieved at pH 10 and that cornstarch acted as a depressant on spodumene [40]. 

**Figure 1:**
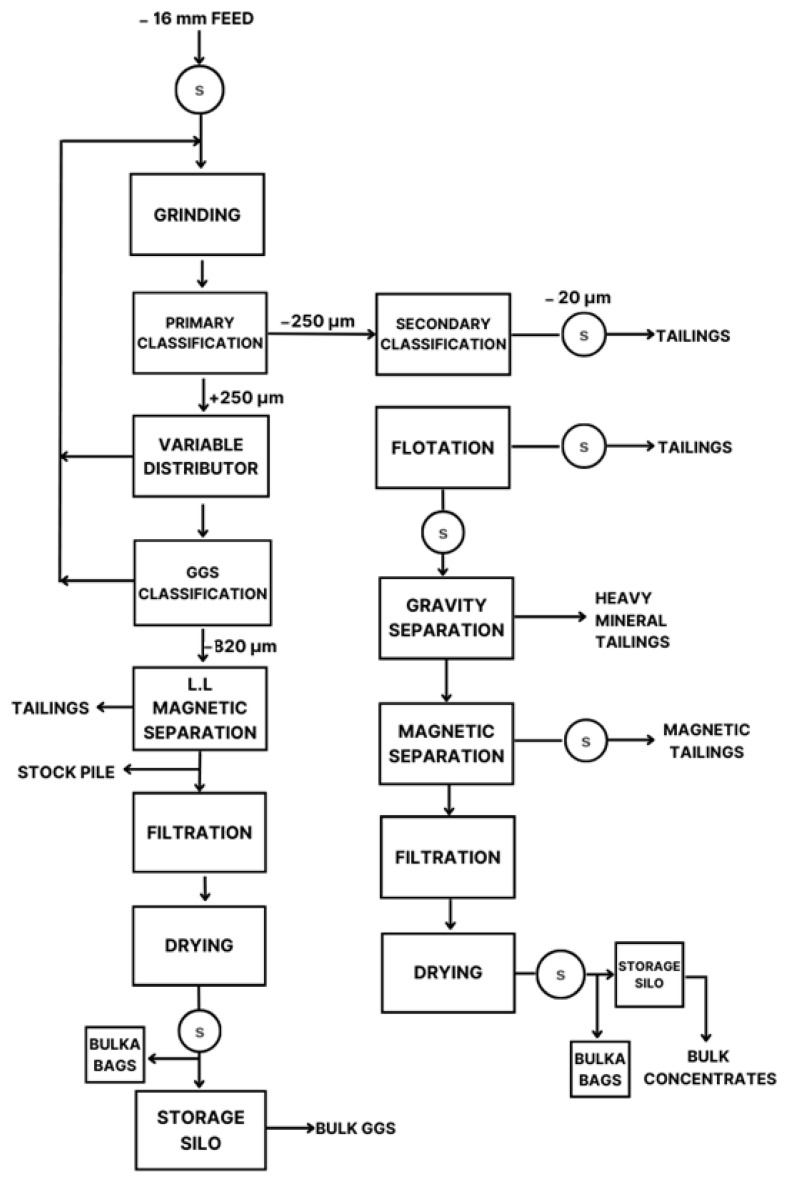
Spodumene processing flow diagram [41].

In 1989, an investigation was carried out with a theoretical approach to extracting tantalum and lithium. In the case of lithium, the research comments that it is extracted from spodumene, a pyroxene mineral, although it also comments on its obtention from other minerals, i.e., petalite, lepidolite, and amblygonite. It proposes a process flow diagram, shown in Figure 1, which includes crushing, grinding, classification, flotation, gravity separation, magnetic separation, filtration, and drying. A concentrate containing 7.6% Li_2_O was obtained ready to be stored [41].

Finally, Menéndez and his collaborators determined the optimal conditions for spodumene concentration by flotation with anionic foam. The studied parameters were collector dosage, rotation speed in the cell, feed flow pH, conditioning time, temperature, solids percentage, and reprocessing stages required to obtain a good product. Through flotation tests, he recovered 96.24% working at a pH of 7.5–9.8, 18% of solids, a temperature of 15 °C, and a conditioning period of 10 min. The rotational speed in the cell was 1040 RPM, and the oleic acid concentration was 1.4 kg/ton. Menéndez concluded that it is not possible to increase recovery by performing more than two cycles in the Cleaner cells [42].

### 2.2. Second Period of Research with New Analytical Techniques (2003–2023)

Despite the various bibliographic research that presented spodumene separation techniques, process flow diagrams, or information on lithium minerals, challenges remain in achieving a more efficient separation of spodumene from gangue ores. Among these challenges were the low selectivity of collectors, the impact of particle size on collector adsorption, the influence of the surface chemical characteristic of the ore, and the effect of metal ions. In addition, it remains unclear what mechanisms are used in the interaction between the surface of the ore and the different reactants used in flotation.

For this reason, most researchers focus their studies on using experimental tests and different measurements to analyze the behavior and performance of the cell as well as the associated mechanisms. Table 4 presents a summary of standard measurement techniques and their abbreviations.

Kwang Soon Moon used X-ray photoelectron spectroscopy (XPS) measurements, contact angle measurements, infrared spectroscopy, and NaOL flotation tests to study how spodumene surface crystal chemistry affects flotation. He found that collector adsorption moves the point of zero charge to a more acidic range by shifting the curve to more negative values, achieving maximum adsorption at pH 8.5. This value coincides with the maximum recovery point in the flotation experiment. In addition, there is a greater contact angle on the surface (110) than on the surface (001). His work concluded that collector adsorption is achieved through chemical adsorption between the anionic carboxylate functional group with the surface (110) due to a greater number of available sites than the surface (001). In this way, surface crystalline chemistry is one of the main factors contributing to the selective flotation of the ore using NaOL as a collector of other aluminosilicate minerals [20].

In 2007, research was conducted on the effect of multivalent metal cations such as Fe^3+^ and Ca^2+^ on spodumene flotation to study their impact on mineral floatability. Micro-flotation tests, zeta potential measurements, and Fourier transform infrared spectroscopy (FTIR) were performed. It was found that the best activation of the iron ion occurs at pH 6–9, while for calcium at pH > 11.3. Regarding dosage, iron works best at low concentrations, close to 35 mg/L, while calcium works at a high dosage of 140 mg/L. Zeta potential measurement of the mineral is positively displaced when activated by these ions. However, the activation is more durable when using iron over calcium. In this way, the research concludes that the floatability of spodumene increases when using Fe^3+^ and Ca^2+^ ions as activators [43]. 

In 2013, another investigation was conducted on the behavior and adsorption mechanisms of the ions mentioned previously on spodumene. The objective was to understand these mechanisms and the behavior on the surface of spodumene (110) using micro-flotation tests and zeta potential measurements. They determined that floatability is poor without these ions. However, after adding iron, it reached maximum floatability at pH 8, and with calcium at pH close to 12, which are comparable to the results of the previous research [43]. They added that the adsorption energies of iron and calcium on the surface of spodumene are −369 kJ/mol and −187.14 kJ/mol, respectively, setting Fe^3+^ as more apt to adsorb, as it presents a higher energy value. They concluded that the flotation of spodumene using anionic collectors is low because the activity of lithium and aluminum on the ore surface is too poor to act as active sites; this is increased by using metal ions. This increase is more pronounced in iron than in other ions [31]. After determining that iron has a greater impact on spodumene flotation than calcium, this element was the focus of research of subsequent works that aimed to study its effects at different concentrations and pH. To achieve this, micro-flotation tests, zeta potential measurements, pyrene fluorescence spectroscopy tests, and XPS tests were performed. They obtained that at a concentration of 1.5 × 10^−4^ M of Fe^3+^, a recovery of 90% is achieved at pH 7.1. From fluorescence tests, spodumene shows greater hydrophobicity and floatability than albite and quartz. Meanwhile, XPS analysis indicated that the chemical adsorption on the material’s surface carries out the collector’s adsorption. They concluded that the greatest recovery of spodumene is achieved at a concentration of 1.5 × 10^−4^ M of Fe^3+^ due to an increase in the hydrophobic characteristic of spodumene in comparison to that of the gangue minerals [32].

In addition to the effect of metal ions, topics such as conditioning with sodium hydroxide (NaOH), using oleic acid, or the impact of grinding types were investigated. For example, NaOH’s effect on flotation and its reaction mechanisms were studied using micro-flotation tests, pilot scale tests, reagent studies, and XPS analysis. The idea was to condition the pulp with NaOH for its subsequent flotation. The results showed that as the dose of NaOH increases, spodumene recovery rises to 70%, while feldspar and quartz reach 20%. On a pilot scale, it was found that even though recovery increases, grade decreases due to an increased in floatability of the gangue ores. Another factor considered was conditioning time; as it grows, recovery decreases. Thanks to XPS analysis, it was observed that NaOH treatment exposes more lithium sites on the surface of the mineral, increasing the adsorption of the collector NaOL. The research mentions that the floatability of quartz remains the same but decreases for feldspar. This occurs because as the species sodium carbonate (Na_2_CO_3_) is produced, it acts as a depressant for the gangue minerals. Although NaOH increases spodumene recovery by exposing more Li sites, it should be noted that the concentrate grade decreased when it was used at a pilot scale [44].

In 2015, another study was carried out to determine the impact of wet and dry grinding on the ore’s crystalline structure and surface properties. They performed micro-flotation tests, X-ray diffraction (XRD) measurements, and scanning electron microscopy (SEM). A higher recovery was observed when wet grinding with a particle size between 38 and 45 µm at pH 8.5. Meanwhile, they determined a greater collector adsorption and (110) plane exposure in wet grinding than in dry grinding. The results concluded that wet grinding allows for better collector adsorption by exposing more planes (110) [45].

**Figure 2:**
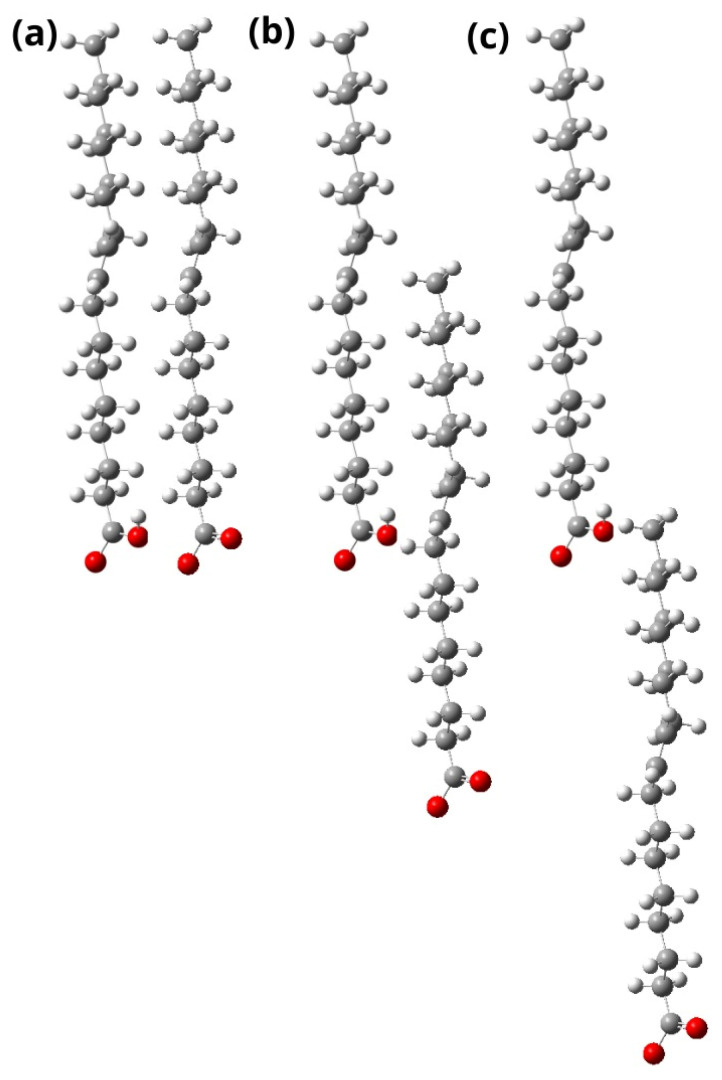
Typical forms of ionic-molecular complexes of oleic acid. (**a**) Head–head interaction; (**b**) Head–middle interaction; (**c**) Head–tail interaction [46]. Red: oxygen atoms, Grey: carbon atoms, White: hydrogen atoms.

In parallel, Fushun Yu and his team aimed to investigate the floatability of spodumene using oleic acid to establish a molecular model of interaction between acid species on the surface of the mineral. They performed micro-flotation tests to measure the surface tension, the hydrophilic–lipophilic equilibrium (HLB), the critical micelle concentration (CMC), and the hydrolysis equilibrium of the oleic acid solution. In addition, using density functional theory (DFT) calculations, they determined how oleic acid species act. The maximum adsorption was observed at pH 8.5 and a concentration of oleic acid of 6 × 10^−4^ M. The solution registers the lowest surface tension in a slightly alkaline range of 8–9; therefore, its hydrophobicity will be greater. The CMC values for ionic oleic acid and molecular complexes explain the maximum recovery obtained at slightly alkaline pH because it has a greater hydrophobic character, achieving a better mineral collection. Chemical analysis illustrates that oleic acid is hydrolyzed to form RCOO^−^ ions at strong alkaline pH and molecular RCOOH at acidic and neutral pH. Using DFT, it was observed that the ion and molecule of oleic acid are associated by van der Waals forces between their heads because the group COO^−^ in molecular complexes reacts with the positive sites of Al on the surface via chemical adsorption as shown in Figure 2. Finally, they concluded that maximum recovery is achieved because the concentration of molecular ionic complexes reaches its maximum at pH 8.5 [46].

**Figure 3:**
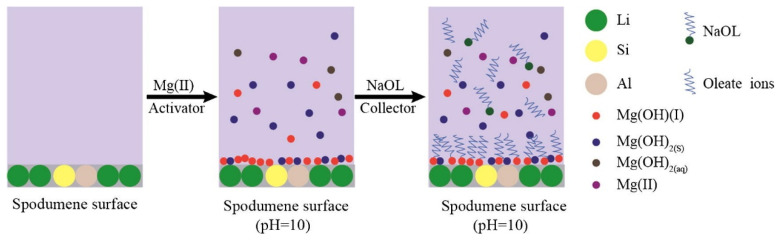
Scheme of NaOL reacting with the different elements on the surface of spodumene after being activated by Mg^2+^ at pH 10 [30].

Although previous articles determined that using iron ions achieved higher recovery and longer-lasting effects than calcium ions, experimental research was conducted on the results of Mg^2+^ and Ca^2+^ using NaOL. The objective was to examine the interaction between these elements, the collector, and the surface of the ore through micro-flotation tests, zeta potential measurements, adsorption amount measurements, FTIR, and XPS. From the micro-flotation tests, it was found that in the absence of ions, spodumene recovery has a maximum of 10% at pH 8, whereas by using a concentration of 120 mg/L of Mg^2+^, the maximum spodumene recovery is 80% at pH 10. Alternatively, using a concentration of 200 mg/L of Ca^2+^, the maximum is reached at 83%, with a pH of 12.5. Regarding the zeta potential, the point of zero charge (PZC) changes slightly and positively displaces the potential. In addition, species distribution diagrams indicate that at the pH where maximum recovery is reached, the concentration of cationic hydroxyl complexes decreases along with spodumene recovery. On the adsorption amount, it was found that it decreased as the pH increased. FTIR analysis found that after adding Ca and Mg ions, hydroxy complexes CaOH and MgOH and precipitates CaOH_2_ and MgOH_2_ were adsorbed. Afterward, when adding NaOL these elements interacted with the portions of RCOO^−^ and R(COO)_2_^2−^ of the collector to form calcium oleate and magnesium oleate, resulting in the flotation of the mineral, as shown in Figure 3. They concluded that adding these elements significantly benefits the flotation of spodumene due to the formation of hydroxy complexes and precipitates that react with the collector, forming new species that adhere to the surface of the mineral and increase its recovery [30].

**Figure 4:**
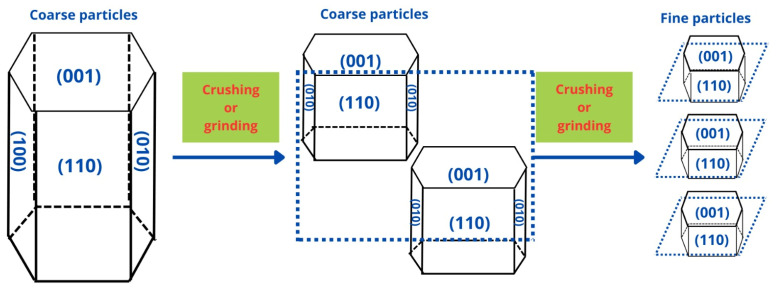
Schematic of the change in the crystalline planes of spodumene as particle size decreases [18].

Particle size of the ore is one of the most relevant parameters in the separation of minerals using flotation, and thus, knowing in detail its impact is key to achieving a good recovery. In China, how particle size affects the chemical properties of spodumene surface crystals was studied. The objective was to elucidate the impact of different sizes on the anisotropic properties of the mineral and its effect on the adsorption of the collector using micro-flotation tests, zeta potential measurements, FTIR, surface energy, broken-bond calculations, molecular dynamics simulations, collector adsorption measurements, and SEM analysis. It was observed that there is a more significant recovery in a size range of 38–45 µm, as well as a greater chemical adsorption of the collector. This NaOL adsorption is attributed to more Al sites in that size range by exposing more planes (110), as shown in Figure 4. The simulation results showed that the collector prefers to bind in a monodentate chelating complex configuration on the plane (110), as its two oxygen atoms bind with the aluminum atom on the surface. In this way, they verified the particle size’s impact [18]. 

In 2016, in parallel, given the problem of low recovery by anionic collectors and low selectivity of cationic collectors, the use of a mixture of collectors was proposed to take advantage of the characteristics of each type. In this line, an investigation was developed on the combinations of anionic and cationic collectors. Dodecyl trimethyl ammonium chloride (DTAC) and NaOL were used to study their selective capacity and other parameters, such as the dosage of calcium chloride (CaCl_2_), NaOH, Na_2_SiO_3_, and temperature. Micro-flotation tests and batch flotation tests were used, where it was found that while the anionic collector is selective, it only reaches a recovery of 55% at pH 8.3. In contrast, the cationic collector achieves a 90% recovery for spodumene and feldspar, demonstrating a low selective capacity. Afterward, mixing both collectors, the molar ratio between NaOL and DTAC that obtained the best results was 9:1, achieving a recovery of 85% of spodumene and 10% of feldspar at pH 8.5. Regarding the rest of the parameters, the mixture presents good results in cold environments, decreasing slightly as temperature rises. On the effect of NaOH on performing alkaline conditioning, an increase in recovery for spodumene and a decrease in recovery for feldspar was achieved. Meanwhile, 1.0 × 10^−3^ M of CaCl_2_ was added to incorporate Ca^2+^. An increase in recovery as pH raised was observed, significantly activating the flotation of spodumene and feldspar. Na_2_CO_3_ was used as a depressant to counteract feldspar activation, reducing feldspar recovery from 95% to 5%, using a concentration of Na_2_CO_3_ lower than 6.0 mM. Finally, micro-flotation tests of artificially mixed minerals were carried out using the collector mixture, achieving a grade of 5.57% and recovery of 71.13% of Li_2_O, while in batch flotation tests, it was found that this mixture reduced collector consumption by two-thirds. They concluded that selective separation is possible in the presence of a combination of anionic and cationic collectors with a molar ratio of 9:10, using NaOH, CaCl_2_, and Na_2_CO_3_, in addition to reducing the collector consumption and increasing the resistance to cold temperatures [25]. The following year, research on this mixture of collectors continued, but this time on the adsorption mechanisms in the spodumene–feldspar flotation system. This study aimed to examine the synergistic interaction and assembly performance between anionic and cationic collectors when mixing, as well as to study their properties and adsorption. In addition to flotation tests, surface tension measurements, adsorption measurements, zeta potential measurements, and FTIR were performed. It was determined that the maximum adsorption occurred at pH 8.5. Regarding the zeta potential, there was not much difference. However, the authors mentioned that the adsorption of NaOL and DTAC is carried out together in the form of an electroneutral combination. FTIR analysis proposed that NaOL physically reacts with the aluminum sites on the surface, and then the DTAC reacts with the anionic collector to form the electroneutral complex. They concluded that the mixed collector shows high selectivity due to higher surface activity and denser molecular arrangement than the collectors, presenting a synergistic effect in the air/water interface. Meanwhile, DTAC promotes the adsorption of NaOL on the spodumene surface. Finally, DTAC forms an electroneutral complex with NaOL to be absorbed on the mineral surface due to the chemisorption of NaOL on the surface [24].

Chemical bonds, surface energy, broken-bond density of the crystal structure, and their influence on flotation separation were also attractive to researchers, but they compared spodumene with another mineral of the silicate group, albite. To study their impact, research was conducted using SEM and XPS. The study determined that the recovery rate increased for both minerals in the presence of Fe^3+^, although to a greater extent for spodumene. Regarding the particle size, albite presented better results working with finer particle size, while spodumene obtained a better result in a range of 38–45 µm, reinforcing the effects observed in previous research. The floatability of spodumene and albite are linked with the number and type of exposed surface atoms, which are affected by the crystal structure and characteristics of the chemical bonds. This research provides a new perspective, establishing that selective grinding should increase spodumene recovery by exposing surfaces where collector adsorption is favorable [19].

During 2018, multiple topics were developed regarding spodumene flotation. An attempt was made to create new collectors using anionic and cationic collectors as a base, while other studies focused their research on obtaining an environmentally friendly process flow diagram.

Previously, it was found that mixing collectors could be beneficial to flotation separation, and experimental research was conducted on a mixture of NaOL and a dodecyl amine (DDA) collector called YAO. The study’s objective was to determine and understand the flotation performance using the new collector through flotation experiments, zeta potential measurements, contact angle measurements, mineral surface adsorption measurements, and quantum chemistry calculations. Preliminarily, tests were performed using the collectors separately, obtaining a low recovery but high selectivity with NaOL, and a high recovery but low selectivity using DDA. Subsequently, a 10:1 molar ratio of NaOL/DDA, with a concentration of 6 × 10^−4^ mol/dm^3^ for the mixture of the new collector, was used, obtaining a recovery of 85% of spodumene and 20% of quartz and feldspar. Due to the zeta potential measurements performed, it was possible to appreciate YAO’s adsorption on the ore’s surface. The contact angle indicated that the collector turns spodumene the most hydrophobic, then feldspar, and to a lesser extent, quartz. Regarding the adsorption amount, the maximum was observed at pH 8.7 for spodumene, quartz, and feldspar. Using computational results, the adsorption energies of YAO on spodumene in different states were calculated. The energies were negative for all four collector states, indicating that YAO adsorption on the mineral surface is possible in all its states. They concluded that a good spodumene recovery could be achieved while remaining selective due to the preferential adsorption of the collector in its four states on spodumene over the rest of the minerals [21].

**Figure 5:**
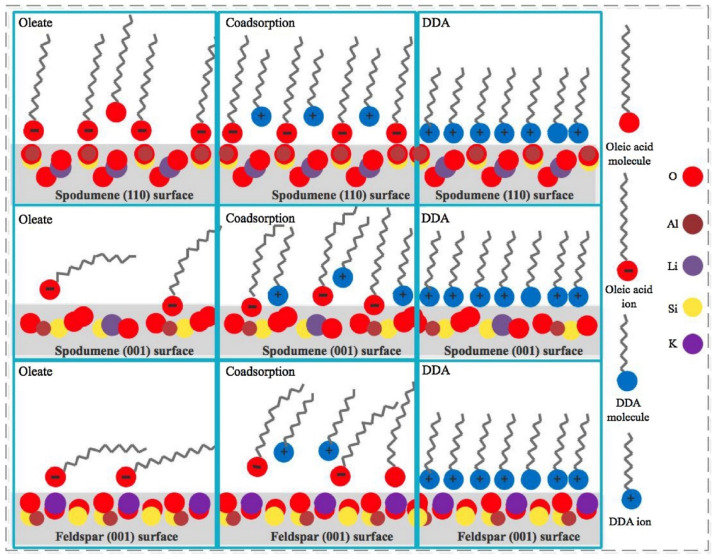
Adsorbed collector structures at surfaces (110) and (001) of spodumene and feldspar [22].

To understand the operation of this mixture of collectors, the same research team published the co-adsorption and association states of the mixture at selected spodumene and feldspar surfaces (110) and (001) to understand their adsorption characteristics. They performed micro-flotation experiments, FTIR measurements, and sum frequency vibrational spectroscopy (SFVS) and used computational tools for contact angle measurements. In the spodumene (110) surface, both anionic and cationic collectors can be adsorbed as a well-packed monolayer, and there is no significant difference when NaOL is mixed with different quantities of DDA. In the case of the spodumene (001) surface, the cation collector can also be adsorbed as a well-packed monolayer. However, in the case of the anionic collector, the adsorption is not as good as in the case of the (110) surface. Regarding feldspar, poor adsorption of NaOL was observed on the (001) surface, which improved as the ratio of DDA increased. Using DFT, it was determined that in the presence of 10% DDA, the cationic collector DDA associates first with the anionic collector NaOL to form a complex. In turn, this gives rise to a more negative charge of the active functional group COO-, and then this group chemically interacts with the Al sites at the spodumene surface, in a phenomenon called co-adsorption that can be observed in Figure 5. They concluded that using a collector mixture formed of 90% NaOL and 10% DDA is favorable enough to separate spodumene from feldspar due to the good adsorption of the anionic collector as an ordered structure that is favored by the presence of the cationic collector. The results of this mixture are explained through DFT due to the adsorption of the stable complex NaOL/DDA due to the co-adsorption states of the collector mixture [22]. 

A different collector mixture was tested using the same anionic collector but replacing the cationic collector DDA with tetradecyl tributyl phosphonium chloride (TTPC). The objective was to study the selectivity of the new mixture and analyze its activity and adsorption mechanisms. This was accomplished using flotation tests, fluorescence spectroscopy, microcalorimetry, and FTIR measurements. Each collector was analyzed separately, finding that the collector TTPC shows high recovery but low selectivity, contrary to what happens with NaOL. Afterward, using a molar mixture of 5:1 NaOL:TTPC, a maximum recovery of 73% spodumene and 6% feldspar at pH 4 was achieved. In parallel, the collector mixture was tested on artificially mixed ores, obtaining a concentrate of 4.6% Li_2_O, with 88% recovery. Spectroscopy analysis found that I_3_/I_1_ values are higher for NaOL/TTPC than NaOL and TTPC individually. Meanwhile, calorimetric results showed higher values of spodumene for the mixed collector and the collectors individually due to its preferential adsorption on spodumene. From FTIR analysis, the adsorption of the new mixture was verified, in addition to being superior to either single collector. Finally, the authors mentioned that obtaining a good recovery and selectivity is possible because the collectors are adsorbed by chemically interacting with each other and then being electrostatically attracted to the spodumene surface. At the same time, only trace amounts are adsorbed on the feldspar surface [23].

Environmental protection and sustainable mining processes were already relevant issues worldwide and not alien to the flotation of spodumene. For this reason, a team of researchers developed a proposal to separate spodumene from mica and feldspar in an environmentally friendly manner while remaining cost-effective. The objective was to provide a process flow diagram to recycle feldspar from flotation tailings. Single-mineral micro-flotation tests were performed to determine particle size, collector dosing, and regulator dosing. They detrmined that the best size ranges were 38–45 µm and 0–19 µm for spodumene and feldspar, respectively. In addition, they determined that a collector dose of 1600 g/t, and a regulator, Na_2_CO_3_ and NaOH dose of 800 g/t, and 300 g/t of CaCl_2_ were required to meet the recovery and concentration demands of Li_2_O. Based on this, they proposed three process flow diagrams to be evaluated in batch flotation tests. The diagram that obtained the best results reached a recovery of 87.24% with a concentration of 6.02%. To recycle feldspar from tailings, they substituted reagents in consideration of environmental health hazards, used magnetic separation to decrease Fe_2_O_3_ content, and obtained a recovery of 6.44%, with a concentration of 11.33% of K_2_O + Na_2_O. They concluded that it is possible to separate spodumene more sustainably with a good lithium mineral recovery while obtaining a concentrate from tailings with high commercial value without harming the environment or causing health hazards [47].

In 2019, in addition to experimental research on flotation tests at fine and coarse particle sizes, the use of lead nitrate (Pb(NO_3_)_2_) as an activator, studying wetting characteristics, evaluating grinding conditions, and testing new collector mixtures, research was conducted with a theoretical approach based on bibliographic reviews to provide relevant information on topics related to spodumene, such as recovery techniques, resources, and the spodumene industry.

Bibliographic reviews allow interested parties to access detailed information on a specific topic, with references that support this information. In addition, they establish similarities or differences in the methodologies, variables, or results obtained, which may be helpful in future research. For instance, a critical review was conducted on the various economic techniques used to benefit lithium minerals. It addressed topics such as the mineralogy of lithium-containing minerals such as spodumene, lepidolite, petalite, amblygonite, zinnwaldite, eucryptite, and others. Afterward, techniques to separate these minerals were described, such as gravity and dense-medium separation, magnetic separation, sensor-based sorting, and flotation. Regarding flotation, it emphasized the surface chemistry of spodumene and lepidolite, their zeta potential, and the effects of chemical pre-treatment. It also mentioned the recovery of valuable by-products and lithium-containing minerals processing plants, such as Greenbushes in Australia, Kings Mountain in the USA, Bernic Lake in Canada, the Bikita operation in Zimbabwe, lithium and tantalum operations in Bald Hill, Australia, and about the future of processing hard rock lithium ore [5]. 

Meanwhile, Huan Li [4] conducted another review to contextualize and serve as a reference point for future researchers. Different deposits and reserves of lithium minerals were reviewed in it, and its extraction from spodumene through various methods, such as acid, alkaline, roasting, autoclave, and chlorination, among others. They concluded that the conventional sulfurous acid (H_2_SO_3_) method has the highest efficiency due to its low cost and advanced technology and estimated it will continue to predominate the spodumene industry. 

Regarding energy use, it mentioned that the alkaline and fluoride routes present lower energy consumption, whereas the roasting or autoclave methods, while increasing consumption, also result in lower impurities in the PLS. Finally, the study mentioned the presence of aluminum or silicon residues after the ion exchange and iron waste product from the fluorine methods and stated they should be appropriately treated for recovery or disposal [4]. 

In parallel, a scientific article with an industrial focus was carried out. It described the lithium industry, market, global resources, and production processes of lithium compounds. The objective was to provide detailed information on lithium processing at an industrial level and then focus on the spodumene phase system, conversion methods, and extraction methods. It concluded that spodumene is the most important source of extraction, which can be perfomed by heat treatment. However, there is not enough information about the process at an industrial level. It suggests that sulfuric acid has been used to treat this mineral, so it would be relevant to develop research using this reagent. It ends by mentioning that the most important factors to consider are the granulometry and the grade of the mineral [48]. 

Grinding is the stage before separation by flotation, so the type of mill used, whether wet or dry, and the particle size will greatly impact the recovery. For this reason, research was conducted on the effects of grinding on spodumene flotation. Three types of mills were evaluated: small corundum mills, large corundum mills, and small nylon mills. In addition, the particle size range was evaluated through grinding tests, acid wash tests, micro-flotation tests, XPS measurements, SEM, and DFT calculations. They obtained the best flotation result in the size range of 75–105 µm for the three types of mills. In the acid washing test, poor results were obtained because washing decreases the concentration of aluminum and metal ion impurities on the surface of the ore, which was validated thanks to the XPS measurements. This coincides with what other researchers have found, alluding to metal ions’ importance in increasing mineral recovery [30,31,32,43]. Indeed, the particle size distribution indicates a greater presence of fine particles in the large conundrum mill, and due to SEM images, the particles were observed to have an elongated, flat, and prismatic shape when using that mill. Finally, DFT found that calcium and iron could be reticular substitutions in spodumene that could act as active sites for collector adsorption. They concluded that the type of mill used will determine the particle size distribution that will enter the flotation stage, but that implementing acid washing could be detrimental to the process, as it decreases the concentration of aluminum on the surface and of metal ions, which are fundamental to increase recovery of spodumene [17]. 

In this line, other researchers have studied the flotation behavior using NaOL and DDA as collectors. However, they were doing so under wet grinding conditions with iron balls and zirconium balls as grinding media. Regarding methodology, they performed zeta potential measurements, XPS, SEM, energy dispersion, X-rays (EDX), and FTIR. For the DDA collector, the recovery was higher when using zirconium balls, while NaOL results were higher when using iron balls. XPS tests indicate that when using zirconium balls, the contents of Al, Fe, Li, Si, and O on the surface change, while with iron balls, the Li content on the surface increases from 9% to 19%, in addition to increasing the Fe content from 0.4% to 2%. Afterward, SEM images revealed that the iron ions served as active spots for NaOL adsorption, increasing recovery compared to zirconium balls. In the case of DDA, iron was found to inhibit flotation. This was verified by adsorption energy measurements, where using the combination of collector and iron balls, it was increased to 8.16 kJ/mol, weakening the interaction between the surface of the mineral and the collector. The opposite occurs with the combination of iron balls and NaOL, where the energy diminishes, strengthening the interaction. In addition, FTIR revealed that iron forms hydroxyl complexes that react with the collector, forming a new species that adheres to the mineral. They concluded that iron balls provided a better result than zirconium balls when NaOL is used as a collector due to the iron content released from the balls due to the abrasion produced in grinding. Meanwhile, in the case of using DDA, it is preferable to use zirconium balls so that iron does not interfere with the recovery of the mineral [14].

In addition to the metal ions previously studied, such as iron, calcium, and magnesium, lead was another element of interest to be used as an activator. A team of researchers performed flotation, adsorption, and atomic force microscopy tests to study the behavior when using Pb(NO_3_)_2_ to incorporate Pb^2+^ ions. DFT calculations were used to analyze the effect of the ion on collector adsorption at the spodumene–aqueous interface. For these tests, the benzo hydroxamic acid (BHA) and Pb(NO_3_)_2_ collectors were used at concentrations of 120 mg/L and 40 mg/L, respectively, recovering 80%. Despite this, in adsorption tests, it was impossible to appreciate a difference in the amount of collector adsorption when in the activator’s presence, or lack thereof. XPS analysis results also corroborated this. AFM testing results indicate that molecules tend to spread on the surface without an activator, whereas when added, they are unevenly dispersed, causing BHA molecules to aggregate irregularly. Finally, using computational results, it was determined that a lack of Pb^2+^ favors a flat adsorption mode between the O^2−^ ion of BHA and the Al^3+^ ion on the surface, with an adsorption energy of −113 kJ/mol, whereas in the presence of Pb^2+^, the adsorption was vertical between two O^2−^ ions of BHA and the Pb^2+^ ion of the surface, with a much higher adsorption energy of −240 kJ/mol. The authors concluded that the addition of Pb(NO_3_)_2_ can activate and improve the collecting capacity of BHA towards spodumene, in addition to providing valuable information to help understand in depth the activation mechanisms of the metallic ions in the presence of anionic collectors in future research [29].

Lev Filippov and his team studied the separation behavior using fine and coarse minerals, sodium oleate as a collector, NaOH and Na_2_CO_3_ as regulators, and calcium ions as activators. The objective was to determine an optimal size range using flotation tests, XRD measurements, SEM, and zeta potential measurements. He obtained better results using a 40–80 μm range than 80–150 μm. This coincides with previous research [45] that determined the highest recovery was achieved in a range of 38 to 45 μm. The zeta potential measurement reaches its most negative point at pH 10. While using the collector becomes more harmful as it reduces the positive charge of the ore surface, adding calcium ions increases recovery for both particle size ranges as the collector adsorbs at negative SiO^−^ sites, forming a relatively dense layer. Regarding regulators, he mentioned that using NaOH increases recovery, while calcium carbonate could be detrimental and decrease performance. To understand the associated mechanism, crystallographic properties were analyzed. The adsorption of NaOL is attributed to the chemical adsorption of oleate in the exposed sites of Al and Si that are generated after breaking the Al-O and Si-O bonds. Calculating the broken-bond strength, the (110) plane is the most favorable as it is the weakest. The 40–80 μm size range has more (001) planes than the 80–150 μm range. This may be because the first range has a higher ratio between the desired planes (110) and the unwanted (100), (010), and (001) planes, so collector adsorption is preferable. They conclude that the finer particle range provides greater floatability when using sodium oleate as it exposes more planes with Al and Si sites than a coarser particle size [49]. 

The wetting characteristics of the mineral were another issue that attracted attention. An experimental investigation on the interaction between water molecules and the mineral surfaces and the adsorption of NaOL and DDA was conducted to provide information on the chemistry of spodumene flotation, surface wettability, the nature of the structure of water, collector organization for selective flotation, and determining the correlation of chemical adsorption of NaOL with the wetting characteristics of the surfaces. They used contact angle measurements, bubble attachment measurements, and SFVS for experimental methodology. They found that all spodumene surfaces are naturally hydrophilic and strongly interact with water molecules and surface atoms. In the presence of a collector, there is preferential adsorption of NaOL on surfaces (110) and (100) over (001) and (010), while for DDA, there is no such preference, as they exhibit a similar hydrophobicity and have same contact angle and bubble attachment time. SFVS verified that the anionic collector is adsorbed in a more orderly manner on the (110) surface than on the (001) surface, as was proposed by Guangli Zhu in his research [22]. They mentioned that the chemical adsorption of NaOL is due to the location of the unsaturated aluminum sites on the surface of the ore, while the adsorption of DDA is mainly performed by electrostatic attraction. Finally, no correlation was found between the spodumene surfaces’ natural hydrophilicity and the collectors’ adsorption [16].

During 2020, a great number of contributions were made to research on spodumene flotation, covering different topics such as new collector mixtures, tests at particle sizes lower than 19 µm, the effect of isomorphic substitutions on the mineral surface, surface dissolution behavior, the NaOH effect, a bibliographic review on chemical characteristics of the mineral surface, the effect of metal ions and collectors, and a study focusing on lithium extraction methods from pegmatites.

That year, tests were carried out on two new collectors, hexyl dipropylamine (HPA) and N-dodecyl iminodiacetic acid (DIDA), to study the behavior of spodumene and feldspar flotation. They performed micro-flotation tests for each separate collector and then as a mixture, alongside zeta potential measurements, FTIR analysis, XPS, and surface tension measurements. At first, the results were not as expected. They obtained a recovery of 0.1% using DIDA, while HPA achieved a recovery of 80% for spodumene and 40% for feldspar at pH 7. Afterward, both collectors were mixed, achieving the best results at an HPA: DIDA ratio of 1:3, resulting in recoveries of spodumene and feldspar of 85.61% and 7.38%, respectively. Regarding pH and concentration, they worked at pH 7 and a concentration of up to 57.14 mg/L, as any higher resulted in a reduced recovery of spodumene and an increased recovery of feldspar. Regarding the zeta potential, the mixture of collectors slightly raises the surface potential of spodumene and feldspar. FTIR analysis suggests that HPA is adsorbed on the surface of feldspar by electrostatic adsorption, while on spodumene, it is achieved via hydrogen bonding. In DIDA’s case, spodumene and feldspar are adsorbed via chemical adsorption. Finally, the mixed collector HPA/DIDA self-assembled in the HPA-DIDA complex that was adsorbed in the spodumene via chemical adsorption and hydrogen bonds, while in feldspar, this occurred only by chemical adsorption. They concluded that although the collectors do not present good results, mixed in a molar ratio of 1:3 HPA:DDA, high recovery and good selectivity are achieved due to the joint adsorption by the mixture of collectors via chemical adsorption and hydrogen bonds in spodumene [26]. 

Like the previous research, Kaiqian Shu used BHA and DDA collectors for their adsorption and interconnection mechanisms in flotation. The objective was to provide information on the selection and design of collectors that are efficient while being environmentally friendly. He performed micro-flotation tests, in situ microcalorimetry tests, zeta potential measurements, FTIR, and XPS tests. Evaluating each collector separately, it was not possible to obtain satisfactory results. However, when working with a molar ratio of 6:1 BHA:DDA, the mixture recovered 88.31% for spodumene and 24.57% for feldspar. The mix of collectors obtained a more significant energy variation in the calorimetry tests and a greater increase in the zeta potential than the separate collectors. Spectroscopy indicated that DDA first forms two types of complexes with BHA via hydrogen bonds. Then, using XPS, it was found that the difference in density of active sites and the different structures of BHA facilitated a greater adsorption of the BHA/DDA complex on the surface of spodumene than feldspar. They concluded that the mixture of collectors at a molar ratio of 1:3 HPA:DDA achieves an efficient separation of spodumene from feldspar, reaching a high recovery and selectivity through the mechanism of preferential adsorption of the complex formed by both collectors on the surface of spodumene [50].

A different study was carried out on a new collector complex of acyloxy-propyl-amine and α-bromododecanoic acid on the flotation of spodumene and feldspar. The study used micro-flotation tests, measured the particle size of the collector, used FTIR, XPS, and estimated the zeta potential of spodumene and feldspar that reacted with or without the collector mixture. Separately, the collectors did not achieve good results; α-BDDA does not present acceptable recovery or selectivity, while DPA only offers good recovery but not selectivity. For this reason, they performed tests for the collectors mixed in a 1:1 molar ratio, reaching a recovery of spodumene and feldspar of 82.14% and 32.48%, respectively, while using a concentration of 14.28 mg/L at pH 4.48. On the particle size of the collectors, it was possible to appreciate a larger size for the mixture of collectors than separately, coinciding with the great recovery achieved, as a larger particle size signified a greater adsorption degree. FTIR tests indicated the existence of a halogen bond between the two collectors, verifying the formation of larger supermolecules. XPS results showed more Al in spodumene before and after collector addition than in feldspar. Zeta potential measurements indicated that the adsorption mechanism of the collector on each of the surfaces is different. For both spodumene and feldspar, the adsorption is due to the electrostatic effect between the NH^3+^ group and the negative surface of the mineral. However, this occurs to a lesser extent in feldspar, as Al and O form AlO_4_, which is much more difficult to break than the AlO_6_ present in spodumene, and as such, the Al sites that can interact with COO^−^ are fewer. In addition, the repulsion between COO^−^ and the negative surface of feldspar is greater than with spodumene. The authors concluded that by combining the collectors DPA and α-BDDA, it is possible to achieve a high recovery percentage while doing so selectively due to the formation of supermolecules that are adsorbed on the surface of spodumene through chemisorption, which occurs less in feldspar due to electrostatic interaction [51].

Le Xu and his team conducted another study on a mixture of an anionic collector made of NaOL and a nonionic one, dodecyl succinimide, in flotation. The objective was to determine its recovery and adsorption mechanisms in spodumene using micro-flotation tests, zeta potential measurements, FTIR analysis, and pyrene fluorescence spectroscopy analysis. They performed the tests with a NaOL/DS molar ratio of 4:1 at pH 8.5 and recovered spodumene and feldspar at 90% and 28%, respectively. Afterward, the mixed collector was tested with mixed ore to resemble what happens on an industrial scale and obtained a recovery and concentration of Li_2_O of 82.67% and 6.53%. Zeta potential measurements indicate that the mixed collector changes the potential curve of spodumene to a greater degree than feldspar. Meanwhile, FTIR analysis results indicate that NaOL/DS presents the same intensities as when only NaOL was used. This suggests that DS did not react with NaOL but adsorbed together on the mineral. Finally, fluorescence spectroscopy analyses show that the mixed collector has a greater collection capacity at the solid/solution interface and can form micelles more easily than collectors alone. In addition, the authors mentioned that NaOL and DS can be adsorbed together on the surface of spodumene thanks to a synergistic effect between them but that, in feldspar, this adsorption is weak. They concluded that the mixture of anionic and nonionic collectors achieves high recovery selectively for both single ore and a mixture. Zeta potential measurements and FTIR indicate that the combination does not generate new compounds and that the adsorption of the nonionic collector occurs through hydrogen bonds, while the adsorption of the anionic collector occurs through chemisorption [52].

As mentioned before, particle size is one of the parameters that greatly influences flotation, and working in an optimal range allows for better flotation and selectivity results [45,49]. To learn more about this variable, researchers studied the effects of shear resistance using a particle size of less than 19 μm to explore the different shear states in floc formation on flotation. They used micro-flotation tests, shear-stirring strength measurements, particle size measurements, and floc microstructure observations. They verified the low recovery in the size range of 0–19 µm and then performed tests in different shear conditions, adding NaOL, which affected the apparent size and flotation rate. An increase in the flotation rate and recovery was appreciated as the shear strength increased. This is due to the aggregation of fine particles that grow the apparent size, forming larger flocs transforming from bimodal to unimodal mode as the shear strength changes. It is mentioned that the size of the flocs fluctuated between 19 and 75 μm, the range where the greatest recovery was observed. They concluded that using fine spodumene in shear agitation with the addition of NaOL significantly improves recovery. Shear influences the apparent size and flotation rate of fine spodumene. Increased shear strength leads to the formation of large, branched flocs but with weak resistance to rupture. However, a very high force leads to fragmentation of large flocs that do not decrease recovery. For this reason, it is necessary to work in a shear strength range of 9.2–11.5 kJ/m^3^ with a constant flotation rate between 0.0194 and 0.0213 s^−1^ [53].

The mineral crystals can contain defects in their lattice structure, including isomorphic substitution by other elements, leading to mineralogical composition variations. In the case of spodumene, substitution primarily occurs with Fe, Mg, and Cr, which will influence the separation performance. To delve further into this phenomenon, a study was conducted on the surface properties of spodumene to determine its impact on wetting and collector adsorption. To achieve this, they relied on micro-flotation experiments, contact angle measurements, FTIR spectroscopy, and computational simulations on the surface of spodumene. Simulated contact angles on surfaces and the spodumene–sodium oleate complex were also studied. Micro-flotation experiments demonstrate that a maximum recovery of 82% is achieved at a high iron concentration at pH 8.5. This suggests that iron enhances the floatability of spodumene, which aligns with previous research findings [31,32]. Contact angle measurements on the (110) surface indicate it is naturally hydrophilic. In the presence of collectors, this surface behavior transitions to hydrophobic. However, there is a preferential adsorption in the presence of high iron content. This was confirmed through FTIR analysis of surface intensity on spodumene surfaces. On the other hand, simulated contact angle results indicated that both surfaces, with or without substitution, are naturally hydrophilic. In the case of iron, the contact angle increases. A completely hydrophilic surface was observed for manganese, and in the presence of chromium, the surface is the same as the unsubstituted surface. Regarding wetting behavior, water distribution on the surface indicates a strong interaction of water molecules with the mineral surface due to the hydrophilic behavior previously mentioned. The presence of metal ions influences this distribution or orientation. Finally, concerning the adsorption of NaOL (sodium oleate), it is enhanced in the presence of substitutions, indicating that the metal ions contribute to distinct interfacial water structures and play a significant role in collector adsorption [15].

Acidic or alkaline treatment alters the surface properties of the mineral by dissolution, changing the quantity, state, and location of elements on the surface. An investigation was conducted on the surface dissolution behavior of spodumene through various experimental tests and measurements using both acidic and alkaline solutions. Analyzing the zeta potential, the surface treated with NaOH showed a decrease in potential, indicating preferential collector adsorption. Meanwhile, the decrease was less pronounced when using HCl, implying lower collector adsorption. The images obtained from atomic force microscopy (AFM) show that spodumene is rough and textured before grinding, making it smoother when in contact with water. When treated with NaOH, it acquires a surface with lower roughness, though not entirely smooth. In contrast, treatment with HCl results in a much rougher surface due to reagent-induced corrosion. It was noted that colloidal substances form and cover the surface after prolonged treatment in an alkaline environment. This leads to a decrease in collector adsorption and, consequently, lower recovery. X-ray photoelectron spectroscopy (XPS) revealed a high percentage of unsaturated Al when treated with NaOH, and a lower percentage when HCl was used, supporting the low recovery obtained with each treatment. Lastly, DFT calculations suggest that Al^3+^ interacts more readily with the collector than other species like Al, Li_2_O, and LiOH. The authors concluded that alkaline treatment using NaOH is more beneficial, as it enhances collector adsorption on the mineral surface, leading to increased recovery. Conversely, acidic treatment with HCl would have a depressant effect on the mineral [54]. 

Since promising results were obtained regarding using NaOH, the same researchers opted to conduct more tests specifically focused on this reagent and its effect on the selective flotation of spodumene from feldspar and quartz. They employed grinding experiments, micro-flotation tests, contact angle measurements, zeta potential measurements, AFM imaging, XPS measurements, point of zero charge (PZC) measurements, and DFT calculations. In this case, a dosage of 750 mg/L NaOH was used, achieving a recovery of 70%. This result confirms the positive findings from measurements in the previous research [54]. No noticeable effect was observed regarding its impact on the particle size distribution. However, the ICP-AES tests found that the concentration of dissolved species for spodumene is Si > Al > Li, and for feldspar, it is Si > K > Al. This indicates that the number of Al sites on the surface of spodumene increases, enhancing the collector adsorption. After calculating the density of broken Al and Al-O bonds for spodumene and feldspar, they determined that the former has more incomplete Al sites and a higher density of broken Al and Al-O bonds than feldspar, which supports the improvement of collector adsorption. DFT calculations indicated that the interaction energy of OH with surface elements of spodumene and feldspar is much greater for spodumene concerning Al, Si, and Li. This suggests that the interaction can occur more significantly in spodumene than in feldspar. They concluded that NaOH does not affect the particle size distribution of spodumene, feldspar, and quartz. However, it is beneficial for separating spodumene from other minerals because it can increase collector adsorption on the mineral surface [55].

The following discussed review was conducted in 2020. It aimed to provide detailed information about spodumene treatments, along with the inclusion of emerging processes. It emphasized that the primary lithium producers are Australia, China, and South America, and their resources are found in saline environments or hard rock pegmatites. In addition to well-known methods such as dense media separation, magnetic separation, and flotation, the review mentioned that fluorination and digestion with caustic pressure could potentially see use soon, but further research is required in these areas. In addition, it emphasized that bioleaching could be a more sustainable option than other thermochemical or mechanochemical methods due to its lower risk and relatively moderate cost [2].

In 2021, research teams continued their pursuit of gaining an in-depth understanding of spodumene flotation and achieving a more efficient process. They studied the behavior of two collectors, PPPDA and α-BDDA. Additionally, the combination of α-BDDA and DDA collectors was investigated. The exploration of metal ions like Fe^3+^ and Ca^2+^ persisted, including Cu^2+^ and Mg^2+^. Further studies were conducted, including a detailed investigation into calcium activation in flotation using anionic collectors and focused research on the adsorption behavior of calcium hydrolysate. Additionally, a study delved into the impact of water hardness on flotation performance.

In the context of collector development during the year 2021, Ruiqi Xie and their research team conducted a study on the behavior and flotation mechanisms of α-bromo dodecanoic acid (α-BDDA) in the flotation separation of spodumene from feldspar and quartz using Ca^2+^ as an activator. The methodology included single-mineral and mixed-mineral flotation experiments, atomic force microscopy analysis, zeta potential measurements, FTIR analysis, XPS, and first-principle calculation. Using a collector concentration of 1.79 mmol/L and 0.51 mmol/L of Ca^2+^ as an activator at pH 7.1, they achieved recoveries of 83.44% for spodumene, 7.83% for feldspar, and 1.38% for quartz. In mixed mineral tests, they recovered 75.57% and a Li_2_O concentration of 5.77%. SEM analysis indicated that mineral surfaces were flat and smooth, but after adding the collector and activator, this characteristic decreased for spodumene and, to a lesser extent, for feldspar and quartz. Zeta potential measurements showed a positive shift after adding Ca^2+^, indicating its adsorption through electrostatic attraction. Adding the collector changed the point of zero charge (PZC), also indicating a chemical interaction. The FTIR analysis results revealed a characteristic peak of the collector, confirming its chemical adsorption on the mineral surface. XPS analyses indicated that the collector and calcium’s main species at pH 7.1 are C_12_H_22_BrO_2_^−^ and Ca^2+^, respectively. This suggests that the potential active sites on activated spodumene for collector adsorption are Li, Al, Si, and Ca. High-resolution spectra showed that Li and Ca were the activated sites for collector adsorption. Given that Li dissolves in solution, the main active sites on activated spodumene for interacting with the collector were those of Ca^2+^. Finally, first-principle calculations revealed that the interaction energy between spodumene and Ca^2+^ was −149.03 kJ/mol, while the interaction energy between BDDA and Ca^2+^ was −365.95 kJ/mol, confirming their chemical adsorption. Furthermore, this adsorption occurred between the calcium site on the surface and the oxygen atom of the collector. The researchers concluded that good flotation results were obtained for both single-mineral and mixed-mineral tests, achieving acceptable recovery and grade. Additionally, the interaction of the collector on the mineral surface was enhanced when using calcium as an activator due to its chemical adsorption on the active calcium sites of the surface and the oxygen atom of the collector [56].

**Figure 6:**
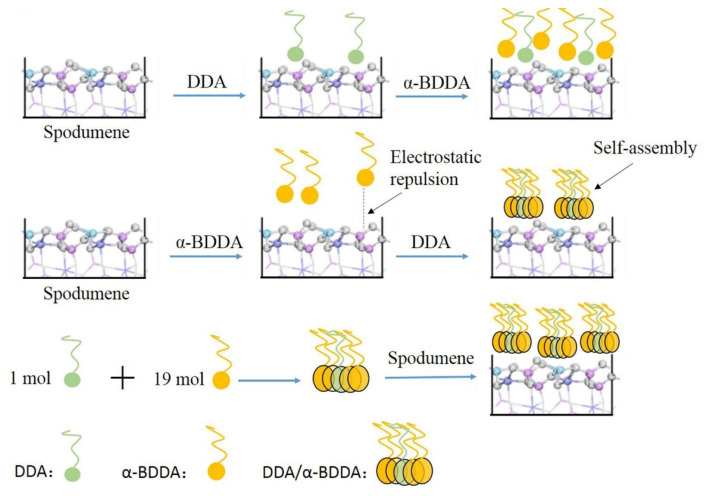
Adsorption mechanism model of α-BDDA and DDA on the spodumene surface [57].

After achieving positive results in their research, the team combined α-BDDA with the cationic collector DDA and studied their behavior and separation mechanisms for spodumene from feldspar and quartz. They employed flotation experiments, atomic force microscopy measurements, contact angle measurements, FTIR analysis, zeta potential measurements, XPS tests, and turbidity analysis. They determined that the optimal molar ratio of DDA:α-BDDA was 1:19, with a concentration of 0.5 mmol/L at pH 7.1, resulting in recoveries of 85.9% for spodumene, 4.48% for feldspar, and 3.92% for quartz. In mixed-mineral tests, they recovered 80%, with a Li_2_O concentration of 6.40% at a lower concentration of 0.43 mmol/L and pH 6. Analyzing the adding sequence of both collectors, it was found that adding the mixed collectors to flotation maximizes recovery due to their self-assembly formation, as opposed to adding them separately. SEM images, contact angle measurements, FTIR analysis, and zeta potential measurements indicated the preferential adsorption of the collector on the spodumene surface compared to feldspar and quartz. XPS analysis revealed that collector adsorption occurred at Al and Si sites. However, given that the most common spodumene surface is (110), the interaction mainly occurred between Al sites and functional groups of the collector. The combined collectors caused significantly higher turbidity compared to separate use, confirming the formation of a self-assembly structure between the two collectors, as depicted in Figure 6. This structure preferentially adsorbed onto Al sites on the spodumene surface, not on feldspar and quartz, due to differences in their crystal structures. In summary, the mixture of collectors achieved a good level of concentration and recovery of spodumene when using the DDA:α-BDDA 1:19 molar ratio pre-mixed before flotation to facilitate self-assembly formation. This increases the hydrophobicity of spodumene due to the preferential adsorption on aluminum sites, unlike the other minerals that retain their hydrophilic character and sink into tailings [57].

An investigation was conducted on a new cationic collector, N-{3-[(2-propylheptyl)oxy]propyl}propane-1,3-diamine (PPPDA), to study its behavior in the separation of spodumene from feldspar and quartz, while considering the influence of metal ions. Flotation experiments, zeta potential measurements, FTIR analysis, AFM imaging, and adsorption quantity measurements were utilized. The flotation tests achieved the optimal separation outcome using a concentration of 0.171 mmol/L of PPPDA and Ca^2+^ at pH 5.2. They attained recoveries of 5.39% for spodumene, 89.31% for feldspar, and 91.7% for quartz, indicating that the collector could be employed in reverse flotation to recover spodumene in the tailings. Regarding metal ions, the study mentions that apart from calcium, magnesium and copper could also be viable options, but iron does not favor this separation as it reduces selectivity. By shifting the potential positively, zeta potential measurements indicated that collector adsorption occurred through electrostatic attraction and hydrogen bonding. AFM images revealed that feldspar and quartz surfaces were flat and smooth, but a significant amount of material was observed upon interaction with the collector. When calcium was added, there were no significant changes in these surfaces. Its surface remained flat and smooth for spodumene, and the addition of the collector led to the formation of material, though to a lesser extent than in the other minerals. In the presence of calcium, material presence decreased, preventing collector adsorption on the spodumene surface. Adsorption measurements confirmed the preferential adsorption of the collector on the surfaces of feldspar and quartz over spodumene. It was also demonstrated that calcium preferentially adsorbs on the latter mineral. The researchers concluded that the collector could be helpful to for reverse flotation, achieving a high recovery of feldspar and quartz and a low recovery of spodumene using calcium as an activator. This is due to the collector’s preferential adsorption on the feldspar and quartz surfaces via electrostatic attraction and hydrogen bonding [27]. 

The effect of magnesium and copper on the separation of spodumene from feldspar and quartz was studied. The objective was to examine the impact of Fe^3+^, Ca^2+^, Cu^2+^, and Mg^2+^ through single-mineral and mixed-mineral flotation, adsorption quantity measurements, FTIR analysis, and molecular orbital analysis. The tests were conducted using BDDA as the collector at a 1.43 mmol/L concentration. Without metal ions, a recovery of 20% was achieved for spodumene, while feldspar and quartz had 0.1% recovery each. Adding an activator concentration of 0.69 mmol/L increased recovery due to the presence of metal ions. In the case of iron, recovery increased but was not selective, whereas the other ions led to a selectively increased recovery. Subsequently, the collector concentration was analyzed for each ion at pH 7 to determine the optimal dosage, followed by assessing the activator dosage. In the cases of iron and magnesium, there are minimal changes in recovery and selectivity as the activator concentration varies. For calcium and copper, selectivity decreases as the concentration increases. It was found that it is better to add Ca^2+^ and Mg^2+^ before the collector, whereas Cu^2+^ and Fe^3+^ are better added after the collector for a more beneficial activator effect. As magnesium and calcium presented better results, they were used for artificially mixed mineral tests. Using calcium, a recovery of 75.6% and a Li_2_O grade of 5.80% were achieved. For magnesium, the results were 76.3% and 5.2%, respectively. The adsorption measurements of these ions increased in the presence of the collector, indicating a synergistic effect, while copper adsorption decreased. In the case of iron, adsorption increased in all three minerals, indicating strong but non-selective adsorption. FTIR analyses revealed different positions, shapes, and intensities of peaks for the metal ions, suggesting collector adsorption on the minerals. However, a significant change in the OH vibration peak of the collector indicated that the adsorption on the activated mineral could be attributed to chemical adsorption. Distribution diagrams of species illustrated that at pH 7, calcium and magnesium predominantly existed as Ca^2+^ and Mg^2+^, while iron and copper existed as various species such as Fe(OH)_3_, Fe(OH)_2_, Cu(OH)_2_, Cu^2+^, and Cu(OH)^+^. Additionally, the collector existed as the monovalent anion C_12_H_22_BrO_2_^−^. Finally, analyses of frontier molecular orbitals indicated that the adsorption of C_12_H_22_BrO_2_^−^, Ca^2+^, and Mg^2+^ was easier on spodumene than on feldspar and quartz. While the adsorption of iron and calcium was complicated, Fe(OH)_3_ preferred reacting with feldspar and quartz, reducing selectivity. Cu^2+^ and Cu(OH)_2_ preferred to adsorb on feldspar and quartz, leading to decreased selectivity of spodumene compared to magnesium and calcium. They concluded that adding calcium, magnesium, and copper metal ions increases the recovery of spodumene when using BDDA as the collector, while iron does not provide sufficient selectivity. Furthermore, there is a synergistic effect in the addition of activators between Ca^2+^ and Mg^2+^, resulting in an efficient recovery in mixed-mineral applications [28].

Subsequently, another investigation focused solely on Ca^2+^ using NaOL as the collector. The objective was to explore the mechanism by which calcium ions activate flotation. They conducted micro-flotation tests, UV spectrum analysis, adsorption capacity measurements, FTIR analysis, and XPS tests. A concentration of 160 mg/L of NaOL was used. Micro-flotation tests revealed that under alkaline pH conditions, recovery increased with a dosage of 100 mg/L of CaCl_2_, reaching the maximum value at pH 11.5. Afterward, at this pH, the activator dosage was evaluated, resulting in an 85% recovery within a concentration range of 80 to 120 mg/L. Additionally, a test was conducted where the mineral was activated and then subjected to washing with deionized water, resulting in a decrease in recovery. UV analyses revealed the presence of nanocolloid particles when Ca^2+^ was mixed with the collector, confirming the formation of a complex between them. Collector adsorption measurements indicated no changes after adding CaCl_2_ and the collector either sequentially or mixed. FTIR analyses confirmed this observation, as the same peaks were observed in all curves. XPS confirmed good floatability in the presence of calcium and the collector, regardless of the dosing order, and collector adsorption remained consistent in either case. Finally, it was mentioned that the colloid or complex formed by Ca^2+^ and NaOL interacts strongly with the surface of spodumene, resulting in favorable flotation outcomes. They concluded that adding calcium can activate the collector’s collecting capacity on spodumene, regardless of the dosage and that the addition sequence is not a determining factor in achieving good recovery. On the other hand, the proposed mechanism by which Ca^2+^ activates flotation is that it forms a complex with the collector, which is firmly adsorbed onto the mineral surface, enhancing its collecting ability [58].

Zhan Yongbing conducted research on the adsorption behavior of the collector NaOL before and after ion activation using the mechanism of hydrolyzed calcium ions on the mineral surface and their effect on collector adsorption. The objective was to determine the calcium mechanism from a microscopic perspective to provide theoretical guidance for developing new reagents. Primarily utilizing micro-flotation tests, computational tools, and XPS tests, the research indicated that the recovery of spodumene increases in the presence of calcium. Species diagram analyses showed that Ca^2+^ predominates up to pH 9, followed by the predominance of the Ca(OH)^+^ species. This component is effective in activating the mineral surface. By studying the effect of water molecules and collectors on the surface, it was determined that the mineral’s surface exhibits strong wettability due to a higher energy interaction of water molecules with spodumene than the collector. The adsorption of calcium ions hydrolyzed on the mineral surface indicated that Ca(OH)^+^ adsorption is greater than that of Ca^2+^. On the other hand, Ca(OH)_2_ is similarly adsorbed onto the surface, involving the binding of calcium ions with oxygen sites on the spodumene and hydroxyl oxygen with aluminum atoms on the mineral surface. The energy of this binding is high due to the strong interaction between the oxygen atoms of the hydroxyl group and the aluminum sites. The study mentions that the adsorption of the collector occurs at calcium sites through bidentate adsorption. Ca(OH)^+^ has a more pronounced effect on increasing collector adsorption compared to Ca(OH)_2_. This is attributed to Ca(OH)_2_ occupying higher positions on the surface, reducing the strength of collector adsorption. Finally, XPS analysis indicates that Ca^2+^ is adsorbed onto the spodumene surface through a reaction with oxygen sites. In addition to this form of adsorption, the hydroxyl group of Ca(OH)^+^ and Ca(OH)_2_ binds to aluminum sites on the mineral surface. The order of strength of calcium ion adsorption on the mineral surface, from least to greatest, is Ca^2+^, Ca(OH)^+^, and Ca(OH)_2_. The study concluded that the presence of calcium ions enhances the spodumene flotation separation due to the higher adsorption energy of Ca^2+^ compared to water molecules on the surface. The existence of other calcium species, such as Ca(OH)^+^ and Ca(OH)_2_, increases the energy of adsorption because their oxygen atoms bond to aluminum sites on the surface, leading to strong interactions. Despite this, Ca(OH)^+^ is the only effective species that activates spodumene flotation, as Ca(OH)_2_ faces an obstacle in adsorption, not reaching the active Al sites and thereby reducing the adsorption strength of the collector [59].

In parallel, a study was conducted to investigate the effect of water hardness on flotation performance. Three types of water and different reagents were evaluated through batch flotation tests. The study examined low-hardness water, medium-hardness water, and high-hardness water extracted from tap water in Guangzhou, Lanzhou, and a mine, respectively. These waters differed in their hardness concentration, Ca^2+^, and Mg^2+^. Notably, higher water hardness corresponds to higher calcium and magnesium ion concentrations. The reagents used included Na_2_CO_3_, NaOH, and CaCl_2_. The flotation results indicated that effective separation could be achieved using both low- and medium-hardness water, although adding reagents was crucial. For the low-hardness water sample, NaOH acted as an activator, while Na_2_CO_3_ acted as a depressant. In the case of medium-hardness water, Na_2_CO_3_ was used to precipitate the multivalent cations in the pulp. However, for the high-hardness water sample, flotation was ineffective even with the addition of the reagents. The study concluded that the quality of the water used strongly influences flotation performance, and using appropriate reagents is essential to achieve favorable results when the water quality is not optimal. Despite this, achieving selective spodumene separation becomes impossible if the water is of high hardness [60].

In 2021, research was conducted on acid or alkaline pre-treatment with NaOH or HCl in spodumene, quartz, and feldspar in order to analyze dissolution behavior and its influence on the separation of spodumene from gangue minerals. They conducted acid and alkaline pre-treatments on agitation and analysis tests through mass spectroscopy by inductive coupling plasma (ICP-MS). Then, they conducted wettability and flotation tests to analyze the buoyancy of spodumene after pre-treatment. They determined that using NaOH or HCl pre-treatment raises the amount of dissolved Al to a higher degree for spodumene. Moreover, they indicated that Si dissolution is faster than Al when alkaline pre-treatment is used, whilst Al dissolution is faster when an acid pre-treatment is performed, showing changes in wettability, zeta potential, and contact angle. Finally, the conclusion is that previous SI dissolution on the spodumene surface has boosted the absorption of NaOL on the surface, increasing the difference in flotation between the mineral of interest and the rest of gangue minerals such as feldspar and quartz [61].

Ultimately, a review was made, detailing an overview of the crucial variables for achieving favorable outcomes in spodumene flotation. This included the chemical characteristics of the surface, the impact of metal ions, and the choice of collectors used in the process. Regarding the chemical characteristics, it discussed the effect of particle size, anisotropic surfaces, surface dissolution, isomorphic substitution, grinding impact, and spodumene’s electronic structure. Concerning metal ions, it highlighted the use of iron as achieving the best results but also mentions calcium, magnesium, lead, and aluminum. Lastly, it addressed three categories of collectors: simple collectors, supramolecular collectors, and mixtures. The first two categories are primarily employed for single-mineral testing or studying chemical characteristics, while the mixture of these collectors is potentially used in industry applications. In summary, it provides detailed information about all the essential aspects needed to initiate research into spodumene flotation [13].

In 2022, emphasis was placed on the pre-flotation stage through research into grinding media such as corundum rods and balls or dry grinding as alternatives to water consumption. Additionally, the development of ultrasonic pre-treatment in spodumene flotation and the option to calcine the mineral prior to flotation was pursued. Furthermore, the effect of metal ions and the activation of calcium ions were revisited.

To increase flotation performance, it is essential to ensure that the pre-flotation stage is as efficient as possible, ensuring that the mineral is in favorable conditions for separation. For this reason, the influence of grinding media, such as corundum balls and rods, on spodumene flotation was investigated. The objective of the study was to determine their effect and the involved mechanisms through grinding experiments, flotation tests, XRD, SEM, AFM, and XPS. In the flotation experiments, a collector mixture of NaOL/DDA at a molar ratio of 6:1 with a concentration of 0.2 mM at pH 8 was used, achieving a spodumene recovery of 80.7% using rods and 40.3% using balls. XRD results indicated that using rods in grinding exposed more of the (110) and (100) surfaces than using balls. SEM analysis suggested a higher aluminum content on the surface of the material ground with rods, and rods also formed sharper edges than balls. Furthermore, AFM images revealed that the minerals treated with rods had a rougher surface than those treated with balls. Finally, XPS revealed that rods expose more aluminum sites than the other grinding medium. The researchers concluded by suggesting that spodumene ground with rods performs better than grinding with balls when using NaOL/DDA as the chosen collector. This difference could be attributed to the cracking and fracturing characteristics of each type of grinding, with the rods exposing a greater amount of (110) planes and aluminum content than the balls [62]. Despite dry grinding being often dismissed due to high energy consumption, it presents certain benefits, such as reduced water consumption and decreased wear on grinding media, making it an attractive alternative. A team of researchers delved into this topic to determine its effect on spodumene flotation. They conducted flotation tests comparing wet grinding and dry grinding methods. They found that wet grinding is 27% faster than dry grinding. Subsequently, in the flotation tests, they achieved recoveries ranging between 60% and 80%, but with low-quality products. They suggested that this could be attributed to the massive activating effect due to prolonged conditioning time. This could lead to a generalized activation of both the target and gangue minerals, rendering most of them hydrophobic and decreasing flotation performance. The researchers concluded that if the activation by the collector during conditioning could be controlled, dry grinding could become an interesting alternative in the future [63].

Another alternative that emerged in 2022 was the implementation of ultrasonic treatment in spodumene flotation, aiming to provide power and intensity to enhance processing rates. Haoran Chu investigated its application in spodumene flotation across various size fractions. The objective was to enhance floatability through this treatment. Chu and the research team conducted micro-flotation tests and collector adsorption studies to compare the effect of the pre-treatment with the traditional process. They also employed inductively coupled plasma optical emission spectrometry (ICP-OES) analysis to study the dissolution behavior of spodumene under different pre-treatment systems. Finally, XPS was employed to analyze the effect of ultrasound on the physicochemical properties of the mineral surface. Three size ranges were utilized, using a NaOL concentration of 200 mg/L in spodumene flotation. The ultrasonic treatment achieved higher recovery in the size ranges of −0.15 to +0.074 mm and −0.074 to +0.0385 mm compared to the traditional method. However, in the size range smaller than −0.0385 mm, no significant difference was observed between the two methods. In the adsorption test, ultrasound was compared with a treatment involving NaOH. Improved floatability was achieved using ultrasound in the first two size ranges, while there was no substantial difference in the third range. ICP-OES analysis indicated that ultrasound promoted the dissolution of surface components by more than double compared to using NaOH with traditional mechanical agitation. Furthermore, XPS revealed a lower amount of Si on coarse and medium-grain minerals surface when ultrasound was employed compared to NaOH. This lower amount of Si allows for increased Al and Li atoms on the mineral’s surface. However, this difference is not as pronounced in the finest size range. The authors concluded that ultrasound treatment holds significant potential as a pre-treatment for spodumene flotation compared to the traditional method across different size ranges. This is attributed to the fact that it enhances the proportion of Al and Li on the mineral’s surface to act as active sites for the collector [64].

Despite extensive bibliographic research on the effects of metal ion activation, the mechanism by which these ions activate the mineral surface is still not clearly understood. For this reason, researchers continue to seek to determine the activation mechanism of Fe^3+^, Mg^2+^, and Ca^2+^ using novel measurements. Researchers employed micro-flotation experiments, the MINTEQ visual model, in situ Attenuated Total Reflectance Fourier Transform Infrared Spectroscopy (ATR-FTIR), and XPS. The flotation tests used concentrations of 0.6 mM NaOL, 0.2 mM FeCl_3_·H_2_O, 0.2 mM MgCl_2_·6H_2_O, and 0.6 mM CaCl_2_. They found that the surface activated by Fe^3+^ recovered 78.4% at pH 6.5, although it decreased afterward. This suggests that iron demonstrates optimal activation in weakly acidic and neutral environments. On the other hand, Ca^2+^ and Mg^2+^ ions achieved recoveries of 89.8% and 94.2%, respectively, at pH 9.4 and 12.1. Analysis using the Visual MINTEQ model indicated that when the highest recovery was obtained with Fe^3+^, the dominant hydrolytic component was Fe(OH)^2+^, which adsorbed onto the surface and acted as the active site for the collector. Above this pH range, the dominant components were primarily Fe(OH)_3_ and Fe(OH)_4_^−^. The former would cover the active sites, weakening the interaction with the collector, while the latter reacted with the surface, making it more hydrophilic. For Mg^2+^ and Ca^2+^, the dominant components were Mg(OH)^+^ and Ca(OH)^+^, which reacted in the aqueous solution with NaOL to form colloidal complexes. Furthermore, the NaOL diagram indicated the presence of a limited amount of oleic acid ions when Fe^3+^ was used at the pH that achieved higher recovery, suggesting a weak activation by iron. In the case of the other two elements, their hydroxyl compounds easily interacted with most of the oleic acid ions to form colloidal complexes in an alkaline environment. It is speculated that in the case of activation using Fe, the adsorption is molecular, whereas with Mg and Ca, it is chemical. ATR-FTIR analyses indicated strong chemical adsorption of NaOL on the surface of Mg^2+^-activated, and Ca^2+^-activated spodumene, suggesting that pre-treatment with metal ions enhances the chemical action of the collector on the mineral surface. XPS analysis indicated that the addition of metal ions could activate spodumene flotation in two ways: by providing more adsorption sites for the collector and by the collector interacting with the metal ions to form micelles in the aqueous solution, which are then adsorbed to achieve spodumene activation. The researchers concluded that metal ions increase the recovery of spodumene by either increasing the number of active sites for collector adsorption or by reacting with the collector in solution to form colloidal complexes that are subsequently adsorbed onto the mineral surface [65].

Xian-Ping Luo aimed to determine the mechanism of calcium hydroxide adsorption on the surface of spodumene to further investigate the use of calcium ions for spodumene activation in flotation. He conducted micro-flotation tests for separate and mixed minerals and employed DFT calculations alongside XPS analysis. In the micro-flotation tests, he added a CaCl_2_ concentration of 5 × 10^–3^ mol/L, recovering about 80% at pH 10 and approximately 90% at pH 12. Afterward, when analyzing the species distribution diagram, a higher presence of Ca(OH)^+^ and Ca(OH)_2_ was observed within that pH range, with Ca(OH)^+^ being the more predominant species in terms of concentration. This indicates that the activation is primarily driven by these components, as evidenced by the increased recovery at that pH. DFT calculations suggest that the activation of calcium ions occurs in two steps: first, the adsorption of calcium onto the mineral surface, and second, the absorbed metal ions serve as active sites for collectors, enhancing the collector’s adsorption intensity. Furthermore, these results suggest that Ca(OH)^+^ adsorption is greater than Ca(OH)_2_. XPS analyses reveal that the collector interacted with Al and Ca atoms on the surface of spodumene. Micro-flotation experiments on mixed minerals revealed that when using a concentration of 1 × 10^−3^ mol/L NaOL and 5 × 10^−3^ mol/L CaCl_2_, a recovery of 71.80% and a concentration of 6.18% was achieved at pH 9 when the concentration of Ca(OH)^+^ increased to 1 × 10^−5^ mol/L. In conclusion, the researchers found that the hydrolyzed components of calcium ions significantly impact the activation and separation of spodumene, enabling a good selective recovery. This is primarily due to Ca(OH)^+^, which exhibits greater adsorption than other species and acts as an active site for the collector [66].

Finally, in 2023, scientific research in spodumene flotation has remained active, with a primary focus on collectors. Weidi Zhang researched the collector N-Hydroxy-9-Octadecenamide (N-OH-9-ODA) and compared it with oleic acid in the flotation of spodumene and albite. The study involved flotation experiments, zeta potential measurements, FTIR, XPS, and first-principle calculations. The flotation tests revealed that, using a concentration of 6 × 10^−4^ mol/L of N-OH-9-ODA, it is possible to achieve a spodumene recovery of 90% and an albite recovery of 50% at pH 10. Meanwhile, oleic acid only achieved 40% recovery for spodumene and 30% for albite, respectively. Regarding zeta potential measurements, when N-OH-9-ODA was added, spodumene exhibited a larger decrease in potential compared to albite. In addition, when adding oleic acid, the change is similar for both surfaces. From the FTIR analysis, in addition to observing the adsorption of oleic acid on spodumene, the adsorption of N-OH-9-ODA can also be seen. XPS analysis indicates preferential adsorption of N-OH-9-ODA on spodumene over albite, confirming the collector’s selective affinity. The researchers concluded that the N-OH-9-ODA collector efficiently separates spodumene from albite at slightly alkaline pH in a selective manner; however, they noted that further research is needed to investigate the mechanism involved in the interaction between the collector and the mineral surface [67].

Zhimin Ma and her team researched the performance of the mixed anionic/cationic collector NaOl/N-lauryl-1,3-propylene diamine (ND13) for separating spodumene from feldspar. The study involved flotation experiments, zeta potential measurements, and FTIR analyses, which led to proposing differences in collector adsorption on the mineral surfaces. Additionally, ultra-microcalorimetric measurements were employed to compare the heat released during the reaction between the collectors and minerals. Finally, contact angle measurements and XPS were utilized further to analyze the hydrophobicity and adsorption mechanisms in more detail. The flotation experiments on individual minerals revealed that NaOL was selective but achieved only a 51.2% recovery for spodumene and 4.7% for feldspar at pH 8. On the other hand, ND13 recovered around 99% when the pH was above 6. Subsequently, by using a molar ratio of NaOL/ND13 of 5:1 and a concentration of 0.4 mmol/L, a recovery close to 80% was achieved for spodumene and 1% for feldspar. A recovery of 82.52% and a concentration of 4.76% were obtained for mixed minerals. The zeta potential measurements indicated that NaOL and ND13 were adsorbed on the mineral surface through chemical and electrostatic adsorption. The FTIR measurements’ results demonstrated that the dominant species of NaOL/ND13 were strongly adsorbed on the surface of spodumene compared to feldspar. Contact angle measurements showed that the NaOL/ND13 mixture enhances the hydrophobicity difference between spodumene and feldspar. The ultra-microcalorimetric measurements revealed that the higher the absolute values of the net reaction heat for spodumene and feldspar reacting with the collectors, the better their separability by flotation. XPS also indicated that NaOL/ND13 underwent a strong chemical reaction with Al sites on the mineral surface, but the interaction of NaOL/ND13 with spodumene was stronger than with feldspar. They concluded that the selective separation of spodumene from feldspar is highly feasible when using the mixed collector at a 5:1 molar ratio, owing to the strong adsorption intensity on the surface of spodumene [68].

Hepeng Zhou and his team researched benzyl arsenic acid (BAA) and NaOL from the perspective of the difference of solidophilic atoms. The aim was to determine the difference between the adsorption reaction of BAA and NaOL on mineral surfaces based on the activity of solidophilic oxygen atoms. The study involved micro-flotation experiments, adsorption capacity tests, computational methods, and closed-circuit flotation experiments using real mineral samples. They evaluated the difference in atomic activity. NaOL can ionize C_17_H_33_COO^−^, which contains two solidophilic oxygen atoms, whereas BAA contains only two solidophilic oxygen atoms in C_7_H_7_AsO_3_^2−^. As such, when BAA interacts with the mineral surface, the binding strength of the solidophilic oxygen atoms to the adsorption sites will be stronger than with NaOL. The micro-flotation results show that NaOL recovered 77% for spodumene and 37% for feldspar, while BAA recovered 87.87% for spodumene and 37.89% for feldspar. Afterward, they conducted tests using a mineral mixture to verify the favorable outcome of BAA, obtaining a recovery of 67.37% and a concentrate with 6.07% Li_2_O content. Adsorption capacity tests indicated that the adsorption capacity of spodumene was lower and did not differ significantly from feldspar when NaOL was used. However, BAA achieved a higher recovery of spodumene and exhibited a greater difference in adsorption capacity between spodumene and feldspar. The adsorption energies of BAA and NaOL on spodumene were −215.32 kJ/mol and −161.83 kJ/mol, respectively, while on feldspar, they were −145.82 kJ/mol and −142.58 kJ/mol. This suggests that the collector’s adsorption is stronger with BAA. From the actual results of the closed-circuit flotation study on real mineral samples, a recovery and Li_2_O concentration grade of 67.82% and 5.13% were achieved with NaOL, and 75.14% and 5.77% with BAA, indicating the feasibility of flotation using BAA. They concluded that compared to NaOL, the solidophilic oxygen atoms of BAA possess a stronger electronegativity, resulting in a higher adsorption capacity on the surface of spodumene and, consequently, a greater collecting ability [69].

In addition to the experimental research, two bibliographic reviews have been conducted during this year. The first one compiled detailed information about the properties of fatty acids and analysis at an industrial level. The review examined the properties of fatty acids, including species distribution in solution, foaming properties, and solubility. Then, it delved into the fundamentals of the oleate–spodumene flotation system, followed by a historical review involving real spodumene minerals. It suggested that due to the mineralogical complexity of new spodumene deposits and their low-grade characteristics, there is a need to enhance collector selectivity. Therefore, research efforts should incorporate steps used in the industry to achieve successful flotation, such as NaOH washing and conditioning with high-density fatty acids [11]. The second publication involved a comprehensive review of global lithium production, resources, reserves, and beneficiation techniques such as dense media separation, froth flotation, magnetic separation, and classification. Moreover, it covered pyrometallurgical methods like sulfation, carbonation, chlorination, roasting, fluorination, autoclave, fluidized bed, and microwave processes. Additionally, it explored hydrometallurgical techniques such as acid digestion, alkaline digestion, and bioleaching. The review also presented a typical process flow diagram. The author’s conclusion highlighted the significance of pegmatites as the primary source of lithium due to the challenges associated with accessing brines and the prolonged treatment times involved. It mentioned flotation as the most used method and emphasized the need to focus on the mechanism associated with adding metal ions and using mixed collectors. Finally, it discussed that despite the limited industrial acceptance of bioleaching, spodumene exhibits high susceptibility to attack by microorganisms, making bioleaching a technique that should be a subject of future research [9].

### 2.3. Third Research Period with Computational Molecular Simulation Method (2011–2023)

As seen in the previous section, a significant amount of research focused on new and mixed collectors, the influence of metal ions, and mineral surface characteristics. These topics are primarily approached through experimental flotation tests and measurements such as zeta potential, FTIR analysis, AFM analysis, and SEM. Despite using these methodologies, the exact mechanism involved in the effect of these variables remains unclear and has not been determined. Thus, further research in this area is required. From 2011 to now 2023, some researchers have investigated using computational software based on quantum chemistry, density functional theory, and molecular dynamics simulations. These approaches are used for measurements and analysis of the behavior of spodumene in flotation cells with collectors and other elements. These tools enable the analysis of behavior at an atomic level with high accuracy, providing a new perspective of analysis that was not feasible using different research methods. In addition, this approach offers the advantage of not requiring experimental procedures, sample analysis, incurring equipment costs, or the use of laboratory reagents.

**Figure 7:**
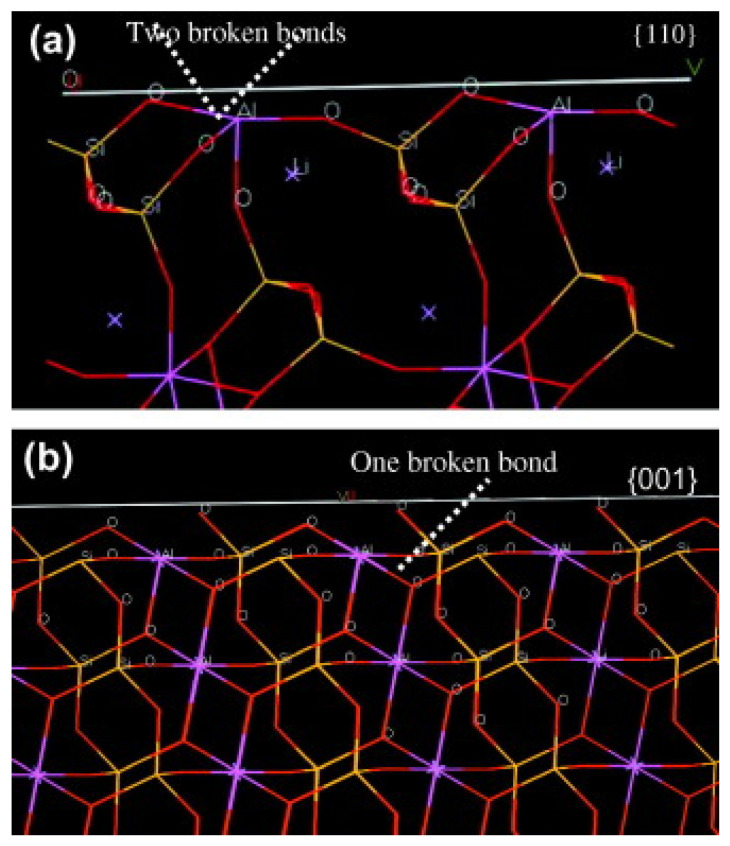
Spodumene surface optimized model. (**a**) Surface (110) (**b**). Surface (001). Color guide: red—O, yellow—Si, pink—Al, and violet—Li [33].

Beena Rai and her colleagues researched the adsorption mechanisms of the collectors NaOL and dodecylammonium chloride (DAC) on various minerals such as spodumene, jadeite, muscovite, and feldspar. They conducted flotation tests, contact angle measurements, and analyses based on molecular dynamics simulations to study the interaction between the mineral surface and the collectors in detail. The simulations were carried out using force field methods, which can model systems containing hundreds of atoms with reagents and minerals. The software used for this purpose was Materials Studio by Accelrys Inc., USA. As spodumene is an anisotropic mineral with a predominant cleavage in the (110) plane, the simulated surface would consist of (110) planes in addition to the basal planes (001). These surfaces can be observed in Figure 7. The (110) plane is more favorable for the chemical adsorption of oleate molecules compared to the (001) plane, as it contains Al atoms with two broken bonds, while the (001) plane has only one broken bond. The adsorption of oleate molecules forms a monolayer on the surface, making it hydrophobic. This phenomenon leads to a well-packed monolayer and, consequently, a higher contact angle, increasing hydrophobicity on the (110) plane compared to the (001) plane. Unlike NaOL, the structural difference between the planes should not affect the adsorption of DAC molecules, as the adsorption is solely due to electrostatic interactions and not the formation of complexes with aluminum sites on the surface. The interaction energy between the two planes and DAC confirmed the previous observations, as the energy was nearly identical. Because jadeite is a mineral similar to spodumene, the interaction energy was also analyzed. The interaction was quite similar, suggesting that the flotation behavior directly relates to the hydrophobicity of the mineral surfaces. As flotation involves spodumene and other minerals, oleate behavior with feldspar and muscovite was analyzed. Comparing the interaction energies for both minerals, it was lower than that for spodumene, indicating that separation would be possible due to the collector’s preferential adsorption. The researchers concluded that molecular modeling calculations can effectively describe the crystalline structure of collector molecules in different silicate minerals. The observations based on molecular simulation models align with experimental findings [35].

**Figure 8:**
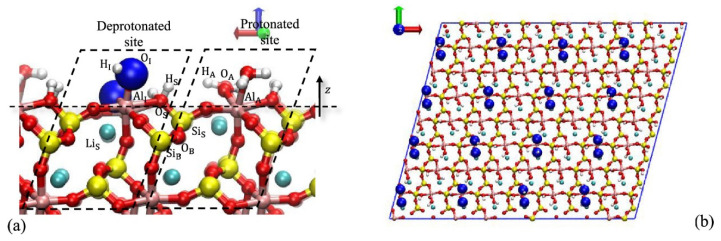
Spodumene surface model of plane (110) with Al, Si, O, H, and Li atoms represented by pink, yellow, red, white, and blue spheres, respectively. (**a**) Lateral view of the surface. (**b**) Top view of the spodumene surface depicted as a box fragment containing 8 × 8 × 3 unit cells [34].

Quezada and Toledo (2021) [33] researched the adsorption of seawater and alkaline earth metal cations on spodumene. The aim was to use DFT to determine the (110) surface and then investigate the interaction between the mineral and the solution through molecular dynamics simulations. Gromacs 5.1.2 was used to conduct the simulations, with SIMD AVX 56 instructions and GPU acceleration to enhance computational speed. The ClayFF classical force field was also employed to model the spodumene–solution interaction. The surface was prepared by cutting the spodumene crystal parallel to the (110) plane, resulting in four broken Al-O bonds and one broken Li-O bond per unit cell. The representation of the spodumene (110) surface can be observed in Figure 8.

Subsequently, simulations yielded the cationic density profile. This indicates that the adsorption of cations is favored as the surface charge of the mineral increases, and it increases even more in the vicinity close to the surface. It was found by analyzing each cation that Li^+^ and Na^+^ are adsorbed more closely due to their small size and high charge density. Mg^2+^ adsorbs away from the surface, between the second and third layers of mineral hydration, forming only outer-sphere complexes. Meanwhile, Ca^2+^ and Sr^2+^ are adsorbed, forming a first layer of inner-sphere complexes in the same position as the spodumene’s second hydration layer and a second layer of outer-sphere complexes between the second and third layers of hydration.

Regarding cation adsorption, the density decreases as the size of alkali cations increases and slightly increases as the size of alkaline earth cations increases. The adsorption maps indicate that Li^+^ and Na^+^ are adsorbed in the corners and crevices of the mineral surface around the anionic centers of AlOH^−1/2^.

On the other hand, K^+^, Rb^+^, and Cs^+^ cannot penetrate the first layer and, therefore, cannot access these corners. Mg^2+^ is adsorbed while completely hydrated and away from the surface, forming a second adsorption layer covering most of the mineral’s anionic and cationic sites. On the other hand, Ca^2+^ and Sr^2+^ form the first layer of cations in the second hydration layer. They conclude that DFT calculations reveal that deprotonated sites have two anionic groups and only one cationic group per unit cell. Below the point of zero charge of spodumene, the anionic collector molecules interact with the surface anionic groups through electrostatic forces. Above the point of zero charge, the anionic collector molecules adsorb onto the single cationic group on the surface, resulting in a more negative zeta potential. MD simulations revealed that the surface adsorption density of each cation, whether alkali or alkaline earth, increases with the surface charge density of spodumene. However, the density consistently decreases as the size of the cations increases. The adsorption maps suggest that in addition to cation adsorption density, the spatial distribution of adsorbed cations is crucial for optimizing flotation through collectors [34].

**Figure 9:**
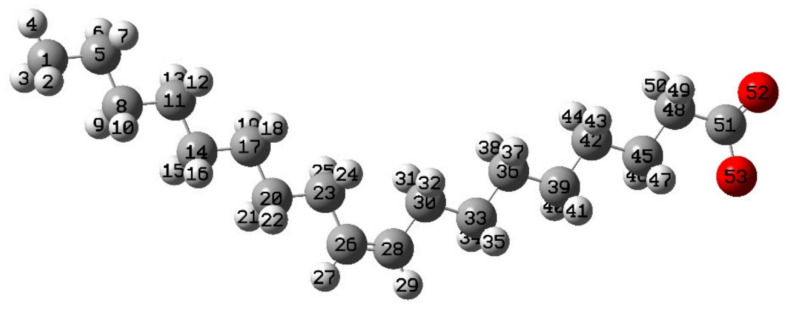
NaOL molecule. The numbers indicate the quantity of atoms. Red: oxygen atoms, Grey: carbon atoms [35].

The following year, Quezada and Toledo, 2021 continued their research, but this time focusing on the impact of seawater and the adsorption of alkali and alkaline earth metals on the adsorption of NaOL. In this case, they utilized the Gromacs 2016.5 molecular dynamics simulation package with SIMD AVX 256 instructions and GPU acceleration to expedite the calculations. The interaction between spodumene and the solution was modeled using the ClayFF classical force field. For the interaction with the collector, a NaOL molecule was added to the simulation box within the liquid phase, as depicted in Figure 9. To complete the simulation in the software, they conducted force minimization, adjusted the system pressure to 1 bar, and set the temperature to 300 K with the ions allowed to move freely. With the simulation ready, they focused on evaluating the role of seawater and cations as barriers and catalysts at pH 8, ensuring that the collector was fully ionized. They found that oleate adsorption onto spodumene is low for all cations, and the limited interaction occurs mainly through cationic bridges, with almost no hydrogen bonding interaction. This is due to the spherical obstacles created by the cations on the surface of spodumene. Furthermore, they suggested that the adsorption of NaOL may be further affected in the presence of seawater. They concluded that the cations form a layer that acts as a barrier, preventing the collector from reaching the spodumene surface. This is because the cations occupy the mineral’s corners and crevices, potentially impacting spodumene’s recovery. This effect could be even more pronounced when seawater is present [35].

## 3. Discussion

Throughout the years, there has been a prevalence of experimental methodology with test flotation and acquisition of measurements with SEM, XPS, XRD, FTIR, or XRF analyses to determine different questions related to spodumene flotation. Said questions are based primarily on determining optimal parameters of flotation, analyzing superficial characteristics, reactive doses, applied collectors or activators, and pre-treatment techniques, among others previously reviewed.

Despite good results, the search for optimizing and satisfying lithium demand is still ongoing. To avoid a waste of resources and time, molecular dynamics simulations are presented as a highly interesting alternative due to the possibility of analyzing the different elements of flotation from an atomic perspective.

Particularly, spodumene and other minerals in interaction with NaOL and DAC collectors have been studied. In this research, (110) and (001) spodumene surfaces were studied, with (110) being the one that gives way to higher spodumene recovery with collectors used at an industrial level and lab collectors such as NaOL and DAC [18,46]. An analysis of simulations of molecular dynamics was made to determine the interaction energy and compare it with measurements of angle of contact obtained in experiments, as well as the pH effect.

The interaction energy results indicate the preferential absorption of the collector on the (110) surface over the (001) one, and it is made preferably on the spodumene over other minerals, making separation feasible. The results obtained from the simulations coincide with experimental results without having to conduct tests in labs, use measurement equipment, minerals, or reagents, saving both time and resources [33].

On the other hand, spodumene flotation has been studied through the use of seawater. The use of seawater has been a highly researched topic recently due to the water crisis that the planet is facing. However, it presents challenges, such as the corrosion of equipment and the different ions that seawater contains, which can negatively affect mining processes [70,71]. Despite these problems, the use of simulations has given way to further study its usefulness with spodumene surface (110), collector molecules NaOL, and the different ions seawater is composed of. Through obtaining the density profile of alkaline metals and cations near the spodumene surface, and thanks to the frequency of interactions between NaOL and the mineral, it can be determined that the use of seawater on spodumene flotation diminishes the absorption of the collector from the surface due to the seawater ions acting as barriers. As a result, it was concluded that no experimental tests, reagents, or excessive resources were needed to use seawater in the reduction of spodumene flotation performance [34,35].

It can be observed that simulations make it possible to represent mineral surfaces, collector molecules, or atoms from activators or reagents, commonly used in pre-treatment in an aqueous medium, obtaining numbers of hydrogen bridges, interactions at minimum distance, density profiles, or absorption maps. Despite its great capacity of representing interactions among elements, its use has been severely limited, which could be attributed to the lack of knowledge on how to conduct these simulations, the time involved in them, or simply lack of equipment. Its limited use could benefit future research and bring valuable information on different phenomena associated with spodumene flotation using this technology. However, efforts to incorporate this methodology must be made, so information can be more easily available, optimizing the process and determining its mechanisms.

## 4. Conclusions

After categorizing multiple investigations into three sections based on the year and methodology used for spodumene flotation, each was reviewed. The studied variable, methodology employed, relevant results, and conclusions were detailed. In this way, valuable information was gathered about how research has been conducted and what techniques have been employed over the years, to provide a theoretical guide and identify gaps for future investigations. The conclusions and implications were as follows:

A noticeable shift in research focus and methodology can be observed. In the initial years, investigations primarily involved experimental tests to achieve the highest possible recovery and concentration. Subsequently, alongside achieving favorable flotation performance, SEM, XPS, FTIR, zeta potential measurements, and adsorption energy measurements were performed to elucidate the mechanisms associated with the interactions between the mineral surface and other elements present in the cell, such as collectors, activators, or depressants. Other researchers have moved away from experimental tests and have employed computational tools such as molecular dynamics simulations to determine the interaction mechanisms of spodumene in flotation.

Software tools, such as Materials Studio and Gromacs, are used for conducting molecular dynamics simulations, enabling an accurate description of the phenomena involved in spodumene flotation. These simulations coincide with experimental results and measurements and offer detailed insights into the interaction mechanisms between the mineral surfaces, ions, and collectors.

The most critical factors in flotation performance were confirmed, such as the chemical characteristics of the mineral surfaces, the use of metal ions as activators, and the collectors used. Concerning the chemical characteristics of the surface, plane (110) presents the best flotation results due to its heightened exposure of active sites for the adsorption of metal ions and collectors. As such, in addition to pre-treatment techniques such as alkaline treatment with NaOH, ultrasonic treatment, different grinding media, or calcination, research should focus on new ways to enhance exposure of this surface.

Activators are another crucial aspect to consider in achieving favorable flotation outcomes. Ions such as Ca^2+^, Mg^2+^, Fe^3+^, and Pb^2+^ have been extensively studied, yielding intriguing results. Among them, Ca^2+^ and Mg^2+^ appear to be the most promising, achieving high recovery rates and demonstrating exceptional selectivity due to their abundant hydroxyl ions at a wide pH range. On the other hand, the ion Fe^3+^ could also be a viable alternative as it provides high recovery rates. However, its selectivity might not be as high.

Therefore, further investigation could explore its potential when combined with other collectors or depressants to sink the gangue minerals in the tailings effectively. Despite this, ions remain a helpful option for enhancing flotation performance.

Nevertheless, careful consideration should be given to their interaction mechanisms with mineral surfaces, the collector choice, depressants, dosage, and pH. Collectors have been studied extensively over the years. The anionic collector NaOL is the most used, but it does not fully meet the current industry requirements, such as low head grades or the diverse mineralogical composition of the species accompanying spodumene.

For this reason, new blends of this collector with cationic collectors have been developed to achieve better selectivity. Simultaneously, new collectors have been synthesized by building upon anionic collectors, showing high recovery and selectivity, but their industrial application is still pending. Furthermore, research efforts should be directed towards efficient collectors in mixed mineral situations. This approach could determine their potential for industrial application, moving beyond laboratory-scale success.

Finally, given the challenges posed by experimental testing and measurements to determine the interaction mechanism between mineral surfaces, ions, and collectors, molecular dynamics simulations could be an interesting alternative in future research. Materials Studio and Gromacs enable the molecular-level understanding of flotation phenomena, making them valuable tools for upcoming investigations. This is especially relevant in synthesizing new collectors, as these simulations could provide a more detailed insight into their interaction mechanisms with spodumene.

## 5. Future Directions

Molecular dynamics simulations are a tool commonly used in fields such as molecular biology or drug design, and lately, they are being incorporated as an alternative for detailed analysis of mineral extraction processes like flotation. As previously discussed, their application was observed in studying the interaction and synergy between the spodumene surface and various collectors, but they still pose several challenges.

One of the challenges lies in the handling and understanding of the software for conducting molecular dynamics simulations. Moreover, careful consideration is required regarding the equipment used to ensure optimal simulation times. Subsequently, meticulous attention is needed for the correct installation of software, such as Gromacs. Once the tools are prepared, proficient management of these software programs becomes crucial to accurately represent the sought-after phenomenon in the simulation. Finally, an analysis of the simulations must be conducted to obtain results describing the interaction between the mineral surface and other elements. In this context, obtaining results like hydrogen bonding, minimum atom distances, density profiles, adsorption energy, or cationic bridging are valuable tools for describing mineral flotation.

For this reason, future research efforts should outline the characteristics of the equipment used for simulations, along with the specific software and version employed, facilitating accurate replication and contributing to future researchers. Moreover, providing detailed information on all steps involved in the simulation process from generating files with mineral surface information to the commands used for extracting results and analyses is crucial. Armed with this comprehensive information, research utilizing molecular dynamics simulations could progress more effectively, extending to other minerals, reagents, considering the use of seawater, or even exploring other processes like mineral leaching.

Ultimately, future investigations should pay attention to industrial operational parameters to offer new alternatives for collectors, particle size ranges, pre-treatment techniques, or environmentally friendly reagents. In this regard, this computational tool gains significance in the development of environmentally friendly mining, eliminating the need for economic- and time-consuming experimental trials to discover alternatives that achieve this objective.

## Figures and Tables

**Table 1:** Number of results by keyword.

Keywords	Number of Results
Spodumene flotation	133
Lithium recovery	6125
Surface characteristics of spodumene	26
Spodumene molecular design	5
Effect of metal ions on spodumene	20
Molecular dynamics of spodumene	28
Total number of search results	6337

**Table 2:** Number of results by search query.

Search Query	Number of Results
Flotation spodumene and molecular dynamics and lithium recovery	3
Flotation spodumene and effect of metal ions on spodumene	14
Flotation spodumene and molecular dynamics	23
Flotation spodumene and molecular dynamics and surface characteristics of spodumene	5
Flotation spodumene and surface characteristics of spodumene	13
Total number of results	58

**Table 3:** Sections with their respective years and number of publications.

**Section**	**Years**	**Number of Publications**
First section	1953–2004	7
Second section	2003–2023	52
Third section	2011–2023	3

**Table 4:** Measurement techniques by name and abbreviation.

Name of Measurement Technique	Abbreviation
X-ray photoelectron spectroscopy	XPS
Fourier transform infrared spectroscopy	FTIR
X-ray diffraction	XRD
Scanning electron microscopy	SEM
X-ray fluorescence	XRF

## Data Availability

Not applicable.
